# Monitoring the expansion of *Dermacentor reticulatus* and occurrence of canine babesiosis in Poland in 2016–2018

**DOI:** 10.1186/s13071-021-04758-7

**Published:** 2021-05-20

**Authors:** Dorota Dwużnik-Szarek, Ewa J. Mierzejewska, Anna Rodo, Katarzyna Goździk, Jolanta Behnke-Borowczyk, Dorota Kiewra, Natalia Kartawik, Anna Bajer

**Affiliations:** 1 Department of Eco-Epidemiology of Parasitic Diseases, Institute of Developmental Biology and Biomedical Sciences, Faculty of Biology, University of Warsaw, Miecznikowa 1, 02-096 Warsaw, Poland; 2 Department of Pathology and Veterinary Diagnostics, Warsaw University of Life Sciences- SGGW, 159c Nowoursynowska Street, 02-766 Warsaw, Poland; 3 Department of Parasitology, Institute of Functional Biology and Ecology, Faculty of Biology, University of Warsaw, Warsaw, Poland; 4 Department of Forest Phytopathology, Faculty of Forestry, Poznań University of Life Sciences, Poznań, Poland; 5 Department of Microbial Ecology and Environmental Protection, Institute of Genetics and Microbiology, University of Wroclaw, 63/77 Przybyszewskiego Street, 51-148 Wrocław, Poland

**Keywords:** *Dermacentor reticulatus*, Abundance, Seasonality, Range, Babesia canis, incidence, Poland

## Abstract

**Background:**

The significance of tick-borne diseases has increased considerably in recent years. Because of the unique distribution of the tick species *Dermacentor reticulatus* in Poland, comprising two expanding populations, Eastern and Western that are separated by a *Dermacentor*-free zone, it is important to conduct studies on the process of tick expansion and emergence of canine babesiosis. The main aim of the current study was to monitor the expansion of *D. reticulatus* populations from spring 2016 to autumn 2018 to determine (1) the actual geographical range of this tick species, and (2) and the seasonal/annual shift in range limits and changes in distance between Western and Eastern populations of ticks (the size of the non-endemic area).

**Methods:**

Ticks were collected in spring/autumn during a 3-year study. From each season and year at least three pairs of sites from the Western and Eastern populations were selected. Then the mean distance between paired sites was calculated for each season and year. We collected and analyzed data from veterinary clinics on the number of canine babesiosis cases treated in the clinic during a whole year (2018).

**Results:**

Accordingly, further expansion of the two *D. reticulatus* populations was recorded, mainly along river basins. Marked colonization of the gap zone was observed, with a mean annual shift in the range of 2.5–10 km and a steadily decreasing distance between the two tick populations. The occurrence of babesiosis in different regions revealed low numbers of cases in Western Poland (19 cases/year) and the gap area (only 7 cases/year) and high incidence (up to 250 cases/1000 dogs) and fatality (total 3.65%) in Central and Eastern Poland. Strong associations were found geographically between tick and babesiosis occurrence and temporally in the seasonal patterns of occurrence of ticks and outbreaks of babesiosis.

**Conclusions:**

We documented the shift in range limits and continued process of colonization of the gap zone accompanied by the emergence of canine babesiosis in the Eastern expansion zone. Updated maps of the distribution of ticks and occurrence of babesiosis in different regions of Poland have allowed us to predict of the emergence of pathogens vectored by *D. reticulatus.*

**Graphic Abstract:**

**Figure:**
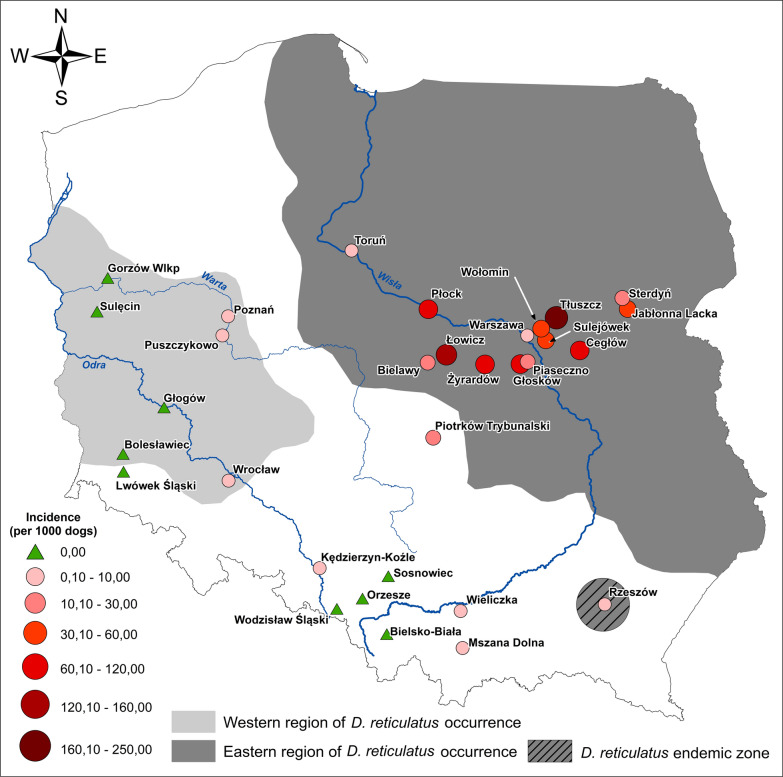
Incidence (per 1000 dogs) of canine babesiosis in veterinary clinics by current range of *D. reticulatus*

**Supplementary Information:**

The online version contains supplementary material available at 10.1186/s13071-021-04758-7.

## Background

The geographical range of the ornate dog tick (*Dermacentor reticulatus*) in Europe is not contiguous and is divided into two metapopulations [1–4]. The Western metapopulation includes areas of France, Belgium, Slovakia, the Czech Republic, Netherlands and Germany [2, 5–8]. The Eastern metapopulation covers Lithuania, Latvia, Belarus, and the eastern and central parts of Poland, along with areas located west of the Vistula River and Russian territory right up to the Ural Mountains [2, 3, 9–11]. Thus, there are two populations of ornate dog ticks in Poland, separated by an area that historically has been free of this tick species (see map in [2]). However, the situation is not static, and in recent decades, geographic expansion of *D. reticulatus* has been recorded in many European countries, including Poland [3, 12].

In Poland (country size: 49°00’–54°50’N and 14°07’–24°09’E**)**, detailed monitoring of the expansion of this tick species was carried out in 2012–2014 [2]. We confirmed the spread of the Eastern Polish population of the tick in a western direction and the expansion of the Western Polish population in an eastern direction. At that time, the easternmost record of the Western population of *D. reticulatus* was near Kościan (Greater Poland Voivodeship, 61 km from the proximate bank of the Oder River and 129 km from the western border of the country); in the eastern area of Poland, the ticks spread to Rawa Mazowiecka and Rzeczyca (Łódź Voivodeship). Finally, the *D. reticulatus*-free zone (in which only negative sites were recorded) was located at the time in the central parts of the country and covered about 150,000 km^2^, from West Pomerania and Pomerania Voivodeships in Northern Poland, to Opole, Silesia, Lesser Poland and Subcarpatia Voivodeships in Southern Poland [2].

The factors responsible for the existence of this tick-free zone (gap) are not known. One hypothesis is that there is a lack of suitable habitats for *D. reticulatus* (wasteland, fallow land, and submerged meadows) in the gap zone [13–15]. Intensive agricultural practices are known to have had a negative impact on tick occurrence and density [16], including the density of *D. reticulatus* populations [15, 17]. However, this cannot be the only reason for *D. reticulatus* expansion into this previously tick-free zone. Rapid expansion of this tick species across the country is likely to lead to the colonization of the gap zone and eventually to a fusion of the Western and the Eastern *D. reticulatus* populations [18]. Determination of the current range of this tick is particularly important due to the epidemiological threat from pathogens transmitted by *D. reticulatus* [19, 20].

One of the most significant pathogens vectored by *D. reticulatus* is *Babesia canis*, the causative organism of canine babesiosis [21, 22]. The prevalence of *B. canis* in *D. reticulatus* is in the range of 1–4% in Poland and varies depending on the region of the country [14, 23]. Ticks infected with *B. canis* were recorded in North Eastern Poland, in Central Poland including in the Eastern expansion zone on the western side of the Vistula River and Eastern or South Eastern areas of Poland [14, 23–26]. Interestingly, to date no *B. canis*-positive *D. reticulatus* ticks have been found in Western Poland [14, 27] (Dwużnik and Kiewra unpublished). Canine babesiosis has spread in Poland over the last 10–15 years [14, 28–32], becoming a serious problem particularly for dog owners and veterinarians in regions where previously it was not known to exist [21, 24, 32].

The drug of choice for treatment of babesiosis is imidocarb dipropionate, which has been used with good efficacy in *Babesia* endemic regions [33, 34] including Poland (such as Imizol injection, Intervet International, Boxmeer, Netherlands). The response to imidocarb is a good indicator for field diagnosis of babesiosis and diagnosis conducted on the basis of clinicopathological findings has a high accuracy rate [35, 36]. Hence, monitoring of the use of Imizol in veterinary clinics is a valuable tool in the study of the epidemiology of canine babesiosis, and reports from local veterinary clinics of the occurrence of canine babesiosis can inform on the distribution of *D. reticulatus*, as this tick species is the main vector of *B. canis* [36, 37]. Veterinary practitioners (VP) are the first to notice the emergence of cases and to implement prevention measures for tick-borne diseases [38]. Babesiosis is a serious problem from a veterinary perspective, not only in endemic areas, but perhaps even more so in locations to which the tick vector has recently spread, where there will be less experience with this disease among both VP and dog owners [39–41]. Knowledge of the current range of *D. reticulatus* ticks is therefore necessary to assess the risk of infection with *B. canis* [36, 42].

The main aim of our study was to monitor the expansion of two Polish *D. reticulatus* populations from spring 2016 to autumn 2018, with a particular focus on the colonization of the gap zone. Regular field collections were performed to determine (1) the actual geographical range of the tick, and (2) the seasonal/annual shift in range limits and changes in size of the non-endemic area. Additionally, we collected data on the distribution and seasonal emergence of canine babesiosis in Poland, including both areas that are endemic (Western and Eastern) and non-endemic for *D. reticulatus* ticks. Finally, we compiled an updated map of the distribution of babesiosis in different regions of Poland, together with the distribution/occurrence of the tick *D. reticulatus.*

## Methods

### Monitoring the present range of occurrence of the *D. reticulatus* ticks

The methods used for tick collection have been described previously in detail [2]. In short, we monitored tick occurrence at two peaks of *D. reticulatus* activity (spring and autumn) for 3 years: 2016, 2017 and 2018 (Additional file 1: Table S1). Locations for monitoring were selected within the previous range of *D. reticulatus* [2] to confirm the persistence of populations, and along the borders of the previous range to detect the spread of *D. reticulatus* (Additional file 1: Table S1). A representative number of locations (22%) were visited two or more times to confirm maintenance of ticks in these new sites, especially in the expansion zones, and to collect ticks for further molecular research. In each location, preferred tick habitats were checked, including fallow land, clearings, wasteland, pastures, river basins, etc. Ticks were collected by dragging using the 1 m^2^ (1.2 m × 0.8 m) woolen blanket, and from the researchers’ clothing and directly from vegetation. After every dragging, scrupulous examination of blankets and clothes was undertaken in order to prevent the transfer of ticks onto the next location. The collection of ticks took place mainly during the morning and afternoon hours. The size of inspected areas varied from 50 m^2^ (endemic areas with high tick abundance) to 1200 m^2^ (expansion zones, gap). Inspected sites were classified as “*D. reticulatus*-positive” if at least one adult tick was collected or as “*D. reticulatus-*absent” if not a single tick was collected from the woolen blanket, researchers’ clothes or vegetation. After confirming the presence of *D. reticulatus* in new locations, new sites were surveyed in a 20–60 km radius, until a “*D. reticulatus*-absent” location was found.

Tick densities were calculated per 100 m^2^. Mean abundance of questing *D. reticulatus* was calculated for every location. Ticks were identified as male/female and preserved at a temperature of −20 °C in the Department of Parasitology, Faculty of Biology, University of Warsaw.

### Distance between Eastern and Western populations of *D. reticulatus* and shift in range

To evaluate the colonization of the tick-free zone (gap), we calculated the distance between two tick populations (Eastern and Western). For this, two sites on the outer border of “*D. reticulatus*-positive” sites (in a particular season) were selected, one from the Eastern and one from the Western population, along the East–West axis: the most eastward located site from the Western population and the most westward located site from the Eastern population. From each season (spring/ autumn) and year of the study (2016, 2017, 2018), at least three pairs of sites from the Western and Eastern populations were selected. Then the mean distance between paired sites for each season and year was calculated (see below).

Additionally, the shift in the tick range was estimated in successive seasons for both populations. For this, we calculated the change in distance between two border “*D. reticulatus*-positive” sites within each population in two subsequent seasons: between sites at the most eastward (for Western population) and most westward (for Eastern population) location at the range border. All distance calculations were based on Google Maps (https://maps.google.com) and the Polish Geoportal website: (https://www.geoportal.gov.pl/). The maps presented in this paper were designed using the ArcGIS (ESRI) geoinformatic software.

### Seasonal abundance of *D. reticulatus* in endemic area

In order to assess the seasonal pattern of *D. reticulatus* abundance, we used data from our long-term study (2012–2016) on tick abundance in several sites in the endemic region of the Eastern population [2, 16, 17, unpublished data]. We also used data collected from seven sites from the current study (2016–2018): Stoski, Kury, Siekierki, Białobrzegi, Korabiewice Owadów, Żyrardów (coordinates in Additional file 1: Table S1). Altogether, data originated from 14 sites [2, 16, 17, unpublished data] and current study.

Tick collections were carried out at these sites from spring 2012 to autumn 2018; in total, mean abundance for each month was calculated based on 4–14 collections (days) each month. Ticks were collected for 6 months of every year, encompassing two tick activity periods: in spring (March, April, May) and autumn (September, October, November). Additionally, in 2017 and 2018, the first appearance of *D. reticulatus* ticks in spring was recorded in one of endemic sites (Stoski or Urwitałt).

### Statistical analyses

The IBM SPSS Statistics v. 21 software package (IBM Corporation) was applied for all analyses. General linear models were used for the analysis of the mean tick abundance, using models with normal errors, incorporating: YEAR (2016, 2017, 2018), SEASON (spring/autumn) and REGION (Eastern, Eastern expansion zone, Western, Western expansion zone).

### The distribution and incidence of canine babesiosis in different regions of Poland in 2018

Our project partners (VP) provided the number of new clinical cases of babesiosis diagnosed in 2018. In two clinics (Tłuszcz, Cegłów, both in *D. reticulatus* Eastern population range), cases were counted prospectively in 2018. Data from other project partners were obtained in 2019 (retrospective survey).

We collected and analyzed data on the number of babesiosis cases treated in the clinic during the whole year (2018) by month, the number of fatal cases due to babesiosis during the whole year (2018) by month, and finally the exact or estimated number of dogs presenting at the clinic during the whole year 2018.

### Definition of cases

Cases were defined as clinical cases of babesiosis when diagnosed by the VP in their practice over the course of a year (2018). Microscopic examination of stained blood smears from capillary beds is rarely performed in babesiosis-endemic regions, due to the need for rapid treatment, so no laboratory confirmation of *Babesia* infection was required. Thus, the collected data represent the exact number of treated clinical babesiosis cases (confirmed or not by laboratory examination) or the number of cases treated successfully with imidocarb dipropionate (Imizol) as recorded in clinics. In total, data from 47 clinics were georeferenced for the construction of a distribution map (Table 5). Cases were assigned to three regions (Eastern, Western and gap) by postcode according to the current *D. reticulatus* range (Fig. 4).

Based on the number of reported treated/fatal cases, several indices were calculated: (1) the annual incidence of canine babesiosis; (2) incidence of canine babesiosis; (3) fatality as % of dogs with fatal outcome; (4) the total number of babesiosis cases/month is presented for seasonal distribution of cases.

## Results

**Fig. 1:**
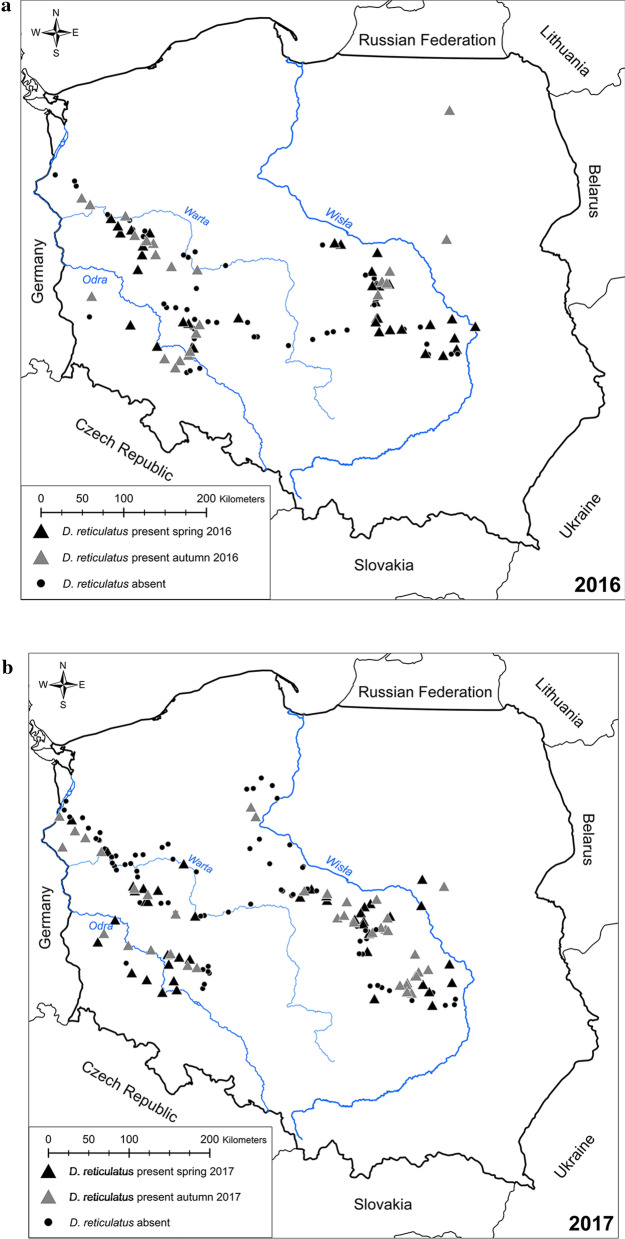

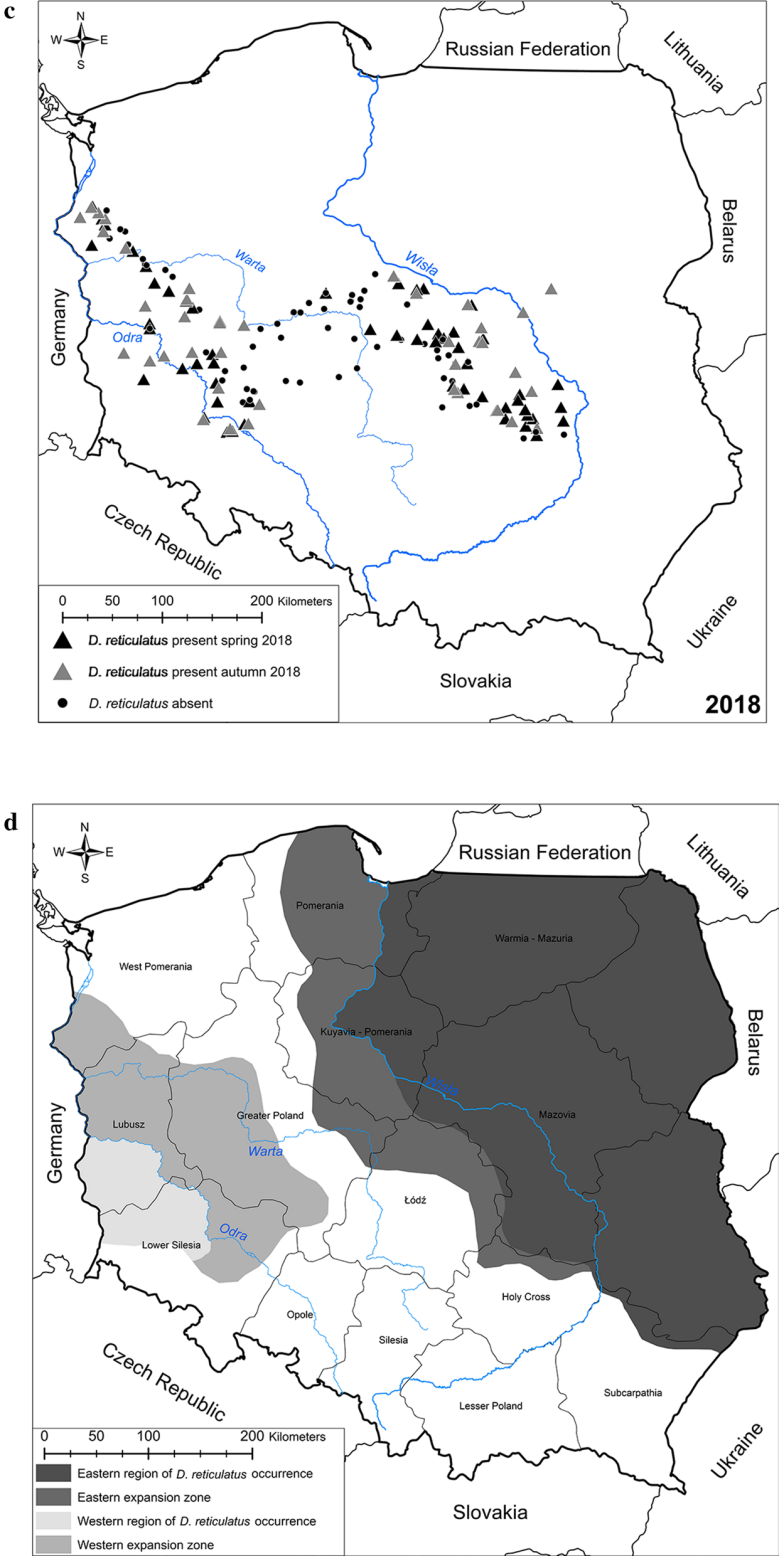
Inspected sites in **a** 2016, **b** 2017, **c** 2018, and **d** current range of *D. reticulatus* in Poland

Altogether, 466 tick collections on 330 sites were performed during the 3-year study (Additional file 1: Table S1), including 123, 168 and 175 collections in 2016, 2017 and 2018, respectively. Among these, 48, 55 and 49 new *D. reticulatus*-positive locations were found in 2016, 2017 and 2018, respectively (Fig. 1a–c). In addition to our previous monitoring in 2012–2014 [2], ticks were found in a number of new sites in eight out of 16 voivodeships: Lower Silesia (30 new sites), Kuyavia-Pomerania (6), Lubusz (20), Łódź (29), Mazovia (34), Greater Poland (25), Holy Cross (1) and West Pomerania (7) (sites listed in Additional file 1: Table S1). 5130 ticks were collected, including 2836 females and 2294 males.

### Current range of the tick *D. reticulatus*

Monitoring of the occurrence of *D. reticulatus* in the area between the Vistula River and the western border of Poland in 2016–2018 revealed seasonal and annual changes in the spatial distribution of this tick species and persistence of the gap zone between the two tick populations (Fig. 1a−c). The current range of *D. reticulatus* in Poland based on the data from our three years of monitoring is shown in Fig. 1d.

### Expansion of the Eastern population in the western, southern and northern directions

Many new *D. reticulatus*-positive locations were found in each season and year of the study in the Eastern expansion zone (Fig. 1a−c): 8, 28 and 24 new sites in 2016, 2017 and 2018, respectively. The main directions of the expansion can be seen in Fig. 1a−c. The majority of the new *D. reticulatus*-positive sites were discovered to the west of previously positive sites (2012–2014), along several river basins, especially along the Bzura River (more than ten new sites) and Pilica River (eight new sites) (both are western tributaries of the Vistula [pol.: Wisła] River) (Fig. 1a−c). Finally, the most westward *D. reticulatus*-positive sites along the Pilica and Bzura river basins were located about 3 km from the riverbeds (close to Tomaszów Mazowiecki and Łęczyca), approximately 120 and 135 km from the Vistula River, respectively (Fig. 1c). In comparison to the previous study (2012–2014; [2].), in which the western border of the Eastern population was found 60 km from the basin of the Vistula River, in the current study the most distant positive site (Sławoszewek, Table 1) was found in spring 2018 as far as 124 km from the Vistula River and 25 km to the north of the Warta River (Fig. 1c).

**Table 1:** Distance of the borders of “*D. reticulatus*-positive” sites within the Eastern population in successive seasons: between sites at the most westward locations

Season	Latitude	Longitude	Season	Latitude	Longitude	Distance (km)
Spring 2016			Autumn 2016			
Bolimów	52.08176	20.1712	Stare Budy	52.07974	20.48895	−21.7
Rawiczów	51.9253	20.1779	Korabiewice	51.95094	20.42969	−17.6
						Mean ± S.E. −19.65 ± 2.05
Autumn 2016			Spring 2017			
Wygoda	51.970261	20.34676	Dębowa Góra	51.90550	20.12974	16.5
Stare Budy	52.079737	20.48895	Dąbkowice	52.06508	19.87396	42.1
Petrynów	51.704731	20.09357	Nowy Kurzeszyn	51.825767	20.27326	18.3
						Mean ± S.E. 25.67 ± 11.00
Spring 2017			Autumn 2017			
Ryków	51.317265	20.76844	Krakowa Góra	51.32158	20.66409	7.28
Gole	52.406193	19.09193	Cetty	52.38489	18.96138	9.18
Dąbkowice	52.065077	19.87396	Oszkowice	52.08343	19.56167	21.5
Dębowa Góra	51.905501	20.12974	Strzyboga	51.91628	20.18895	4.2
						Mean ± S.E. 8.44 ± 6.90
Autumn 2017			Spring 2018			
Cetty	52.384885	18.96138	Sławoszewek	52.3935	18.18320	52.9
Oszkowice	52.083434	19.56167	Chwalborzyce	52.0681	18.83970	48.3
Strzyboga	51.916276	20.18895	Nowe Rowiska	51.90006	20.12697	4.62
						Mean ± S.E. 35.27 ± 20.44
Spring 2018			Autumn 2018			
Sławoszewek	52.3935	18.18320	Skoki	52.39507	19.51830	−90.7
Chwalborzyce	52.0681	18.83970	Słomków	51.95529	19.98709	−79.6
Teofilów	51.5242	20.19500	Tomaszów Mazowiecki	51.52180	20.05790	9.53
Rawa Mazowiecka	51.75236	20.24912	Łochów2	51.74892	20.07967	11.8
						Mean ± S.E. −37.24 ± 47.91
						TOTAL ± S.E. 2.50 ± 24.76

Expansion of the Eastern population was also observed in a southerly direction: in spring 2016 the most southward *D. reticulatus*-positive site was found near Trębowiec Krupów in Holy Cross Voivodeship (the first positive site in this voivodeship). Ticks were also collected at this site in subsequent seasons and years of the study (Fig. 1a−c; Additional file 1: Table S1).

Northward expansion was confirmed by the identification of new *D. reticulatus−*positive sites in Kuyavia−Pomerania Voivodeship, with the northernmost site discovered at the bank of the Brda River (a western tributary of the Vistula River), approximately 25 km westward from the Vistula River and about 20 km to the north of Bydgoszcz (Fig. 1b).

### Expansion of the Western population in eastern, southern and northern directions

With regard to the Western expansion zone, ticks were detected in 26, 30 and 39 sites in 2016, 2017 and 2018, respectively. Altogether, *D. reticulatus*-positive locations were found in five voivodeships: Lubusz (19 sites), Lower Silesia (27), Greater Poland (33) and West Pomerania (16), many more than in the previous monitoring period (2012–2014; [2]).

In each voivodeship, *D. reticulatus*-positive locations were discovered mostly in close proximity to the riverbeds (Fig. 1a−c). Expansion in an easterly direction along the Barycz River was confirmed by 13 new positive locations. Expansion in a southerly direction occurred along the Bystrzyca and Oder [pol.: Odra] rivers (Fig. 1a−c). Expansion to the north was noted along the Obra River (11 new *D. reticulatus*-positive sites situated along the river basin). The most eastward tick-positive sites were detected in the first year of current monitoring: located in Greater Poland Voivodeship about 71, 88 and about 200 km from the Barycz River, Oder River and the western border of the country, respectively (Fig. 1a).

In the north, a new limit to the distribution of *D. reticulatus* was a positive site recorded in West Pomerania Voivodeship, only 30 km to the south of Szczecin, less than three km to the Oder River (Fig. 1b−c). In the south, the border was recorded in Lower Silesia Voivodeship, in close proximity to Wrocław in the Bystrzyca River basin, close to the western bank of the Oder River (Fig. 1c).

### Seasonal distance of expansion/regression

In addition to the marked expansion of the range of *D. reticulatus* across the 3-year period, we also recorded dynamic seasonal changes to the limits of tick distribution, and therefore we calculated the distances for seasonal shifts of the borders of tick ranges: expansion or regression distance for both tick populations.

### Eastern population

Distances were measured between the two closest positive sites in successive seasons, localized along the main axis of the shift (East–West), for at least two pairs of sites following each summer (from spring to autumn) and winter (from autumn to spring). Mean distances are presented in Table 1. Generally, the individual distances of expansion for site pairs (shift in the range causing expansion outside current range) ranged between 4 and 53 km, and expansion was recorded mostly following winter: new sites were found outside the current range in spring 2017 and 2018 but also in autumn 2017. However, in autumn 2016 and 2018, positive sites were found within the previous range, causing “regression” in the range from about 21 to 91 km. Thus, the mean distance of expansion/regression for subsequent seasons ranged between −37 km and +35 km, and overall mean expansion distance was +2.50 ± 24.8 km/season.

### Western population

The mean between-season distances for sites in Western Poland are presented in Table 2. Generally, individual distances for expansion of site pairs (shift to the east in the range causing expansion outside the current range) ranged between 4 and 65 km, and expansion was recorded following the summer of 2016 and winter of 2016/2017, and also for two site pairs following the winter of 2017/2018 and the following summer 2018. However, in autumn 2017 and spring 2018, positive sites were found within the previous range, causing ‘regression’ in the range from about 8 to 31 km. Thus, the mean distance for successive seasons ranged between −16 km and +34 km, and the overall mean distance of expansion over the three year study was +10.15 ± 15.8 km/season.

**Table 2:** Distance of the borders of “*D. reticulatus*-positive” sites within the Western population in successive seasons: between sites at the most eastward locations

Season	Latitude	Longitude	Season	Latitude	Longitude	Distance (km)
Spring 2016			Autumn 2016			
Skwierzyna	52.61080	15.52440	Zamyślin	52.64594	15.78013	17.8
Kuźnica Zbącka	52.23180	16.10573	Grodzisk Wielkopolski	52.23941	16.34532	16.4
Obra	52.06911	16.04106	Bonikowo	52.11618	16.63196	40.8
						Mean ± S.E. 25.00 ± 10.53
Autumn 2016			Spring 2017			
Baczyna	52.74998	15.13779	Janczewo	52.76611	15.34509	14
Zamyślin	52.64595	15.78013	Sobota	52.66563	16.75029	64.6
Bonikowo	52.11619	16.63196	Śrem	52.08752	16.98591	24.4
						Mean ± S.E. 34.33 ± 20.18
Spring 2017			Autumn 2017			
Janczewo	52.76611	15.34509	Gorzów Wielkopolski	52.76383	15.23103	−7.7
Chojna	53.12995	14.42094	Banie	53.097498	14.65297	−16.00
Kościan	52.10445	16.63240	Śrem	52.08753	16.98591	−24.4
						Mean ± S.E. −16.03 ± 5.58
Autumn 2017			Spring 2018			
Chojna	53.12995	14.42094	Banie	53.09980	14.66880	16.9
Rów	52.97834	14.72285	Tarnowo	52.99290	14.82220	6.85
Niezgoda	51.51415	17.04281	Wierzchowice Wielkie	51.55975	16.60470	−30.8
						Mean ± S.E. −2.35 ± 18.97
Spring 2018			Autumn 2018			
Biedaszków Mały	51.39967	17.09867	Czeszów	51.37780	17.24220	10.3
Psary	51.19400	17.03333	Poligon Cienin	51.20000	17.08910	3.95
Trzciel	52.371869	15.86673	Chmielinko	52.40400	16.16590	20.6
						Mean ± S.E. 11.62 ± 5.99
						TOTAL Mean ± S.E. 10.51 ± 15.76

### Distance between the Eastern and Western populations of *D. reticulatus*

Because of the recorded expansion of both tick populations, as described above, we also calculated changes in the width of the gap zone, by calculating the distances between pairs of *D. reticulatus* positive sites, one from the Eastern population, one from the Western one.

The width of the tick-free area was estimated by the distance between the two monitored *D. reticulatus* populations. The mean width for all seasons was 206.25 ± 17.17 km (Table 3). Generally, a shorter mean distance between the two regions was recorded in spring compared to autumn (Table 3). The longest mean distance between populations was observed in the first year of monitoring, in autumn 2016 (239.5 ± 15.50 km) (Table 3). The shortest mean distance between Western and Eastern populations of *D. reticulatus* was recorded in the last season of monitoring, spring 2018, only 162.67 ± 28.22 km (Table 3). The shortest distances between two particular locations from the Eastern and Western tick populations were noted in spring 2017 and spring 2018: only 150 km between Słonawy (Western population) and Katarzyna (Eastern population) and 156 km between Trzciel (Western population) and Sławoszewek (Eastern population) (Table 3). The longest distance (283 km) was recorded in spring 2016 between Wiszna Mała 2 and Trębowiec Krupów (Table 3).

**Table 3:** Distances between the two tick populations (Eastern and Western)

Season	Western population	Latitude	Longitude	Eastern population	Latitude	Longitude	Distance (km)
Spring 2016	Konin	52.47340	16.22720	Gostynin	52.39550	19.51830	223.00
	Odolanów	51.57530	17.82900	Inowłódz	51.53160	20.22590	167.00
	Wiszna Mała2	51.23710	17.05790	Lubiszów Kolonia	51.42360	20.28240	225.00
	Wiszna Mała2	51.23710	17.05790	Trębowiec Krupów	51.17530	21.07630	283.00
							Mean ± S.E. 224.50 ± 29.50
Autumn 2016	Bonikowo	52.116186	16.63196	Puszcza Mariańska	51.970261	20.34676	255.00
	Biedaszków Mały	51.399664	17.09868	Nowy Kurzeszyn	51.825765	20.27326	224.00
							Mean ± S.E. 239.5 ± 15.50
Spring 2017	Słonawy	52.66564	16.75028	Katarzyna	52.31615	18.88637	150.00
	Śrem	52.08752	16.98591	Dąbkowice	52.06508	19.87396	198.00
	Kowaliki	51.60618	16.91092	Petrynów	51.70473	20.09357	220.00
	Brzeg Dolny	51.26023	16.69593	Podkońska Wola	51.17607	20.20582	245.00
							Mean ± S.E. 203.25 ± 29.25
Autumn 2017	Lubiczyn	53.12995	14.42094	Koronowo	53.31464	17.96716	237
	Gorzów Wielkopolski	52.76383	15.23103	Osielsk	53.20956	18.05884	195.00
	Nowy Tomyśl	52.30958	16.11307	Cetty	52.38489	18.96138	193.00
	Kościan	52.10445	16.63240	Oszkowice	52.08343	19.56167	200.00
							Mean ± S.E. 206.25 ± 15.38
Spring 2018	Trzciel	52.37187	15.86673	Sławoszewek	52.39350	18.18320	156
	Śrem	52.08880	16.98710	Chwalborzyce	52.06810	18.83970	127.00
	Biedaszków Mały	51.39966	17.09868	Kaczka	51.55181	20.03962	205.00
							Mean ± S.E. 162.67 ± 28.22
Autumn 2018	Chmielinko	52.40400	16.16590	Dębniaki	52.55046	19.18276	205.00
	Śrem	52.08880	16.98710	Słomków	51.95529	19.98709	203.00
	Czeszów	51.37780	17.24220	Tomaszów Mazowiecki	51.52180	20.05790	196.00
							Mean ± S.E. 201.33 ± 3.56
							TOTAL Mean ± S.E. 206.25 ± 17.17

### Comparison of the mean abundance of *D. reticulatus* between four regions (Western and Eastern endemic areas with their expansion zones)

**Fig. 2:**
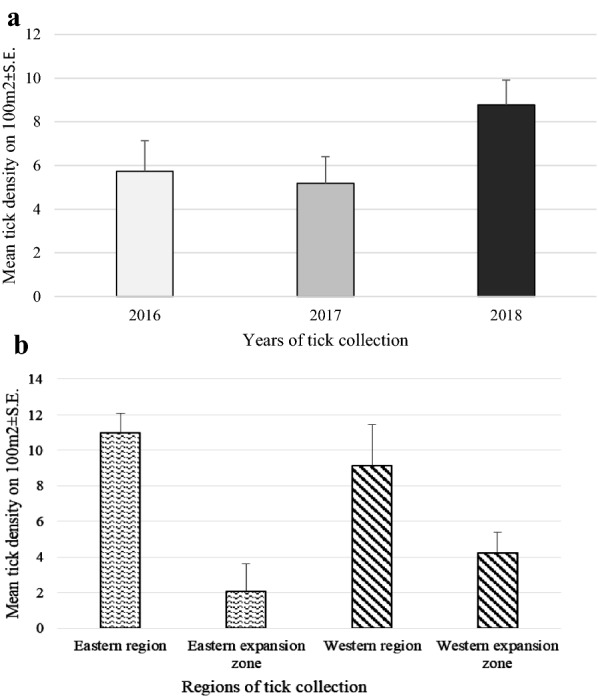
Mean tick abundance **a** by year, **b** by region

The density of ticks per 100 m^2^ was determined at each site (Additional file 1: Table S1) and compared between seasons, years and four regions. Overall mean abundance of *D. reticulatus* was similar in spring and autumn (7.13 ± 1.13 and 6.78 ± 1.52, respectively) (NS). There was a significant difference in mean abundance of *D. reticulatus* between years, mean density being highest in 2018 and lowest in 2017 (8.97 ± 0.1.25 and 4.53 ± 1.50, respectively) (main effect of YEAR on abundance of ticks: *F*_2, 272_ = 2.59, *P* = 0.07) (Fig. 2a). Significantly higher abundance was observed in both known *D. reticulatus* endemic regions in comparison to expansion zones (main effect of REGION on abundance of ticks: *F*_3, 272_ = 10.26, *P* < 0.0001) (Fig. 2b).

Depending on the year and season of the study, mean abundance was highest in the Eastern or Western endemic regions, but generally was lowest in the Eastern or Western expansion zones (YEAR × REGION × SEASON on abundance of ticks: *F*_6,272_ = 2.24, *P* = 0.04) (Table 4).

**Table 4:** Mean tick density by year x season x region

Year	Season	Region	Mean ± S.E.
2016	Spring	Eastern region	5.59 ± 2.52
		Eastern expansion zone	2.09 ± 4.17
		Western region	23.67 ± 7.80
		Western expansion zone	4.65 ± 3.06
	Autumn	Eastern region	15.80 ± 4.17
		Eastern expansion zone	1.50 ± 11.03
		Western region	4.05 ± .5.52
		Western expansion zone	1.72 ± 3.06
2017	Spring	Eastern region	12.88 ± 2.41
		Eastern expansion zone	1.16 ± 3.19
		Western region	5.03 ± 5.52
		Western expansion zone	2.01 ± 2.68
	Autumn	Eastern region	5.08 ± 2.76
		Eastern expansion zone	0.93 ± 3.68
		Western region	5.38 ± 7.80
		Western expansion zone	3.74 ± 3.06
2018	Spring	Eastern region	13.29 ± 2.30
		Eastern expansion zone	3.92 ± 2.60
		Western region	8.34 ± 4.50
		Western expansion zone	2.98 ± 2.35
	Autumn	Eastern region	19.85 ± 3.33
		Eastern expansion zone	0.25 ± 4.50
		Western region	13.05 ± 4.93
		Western expansion zone	10.07 ± 2.68

### Seasonal abundance of *D. reticulatus* in the Eastern population area

**Fig. 3:**
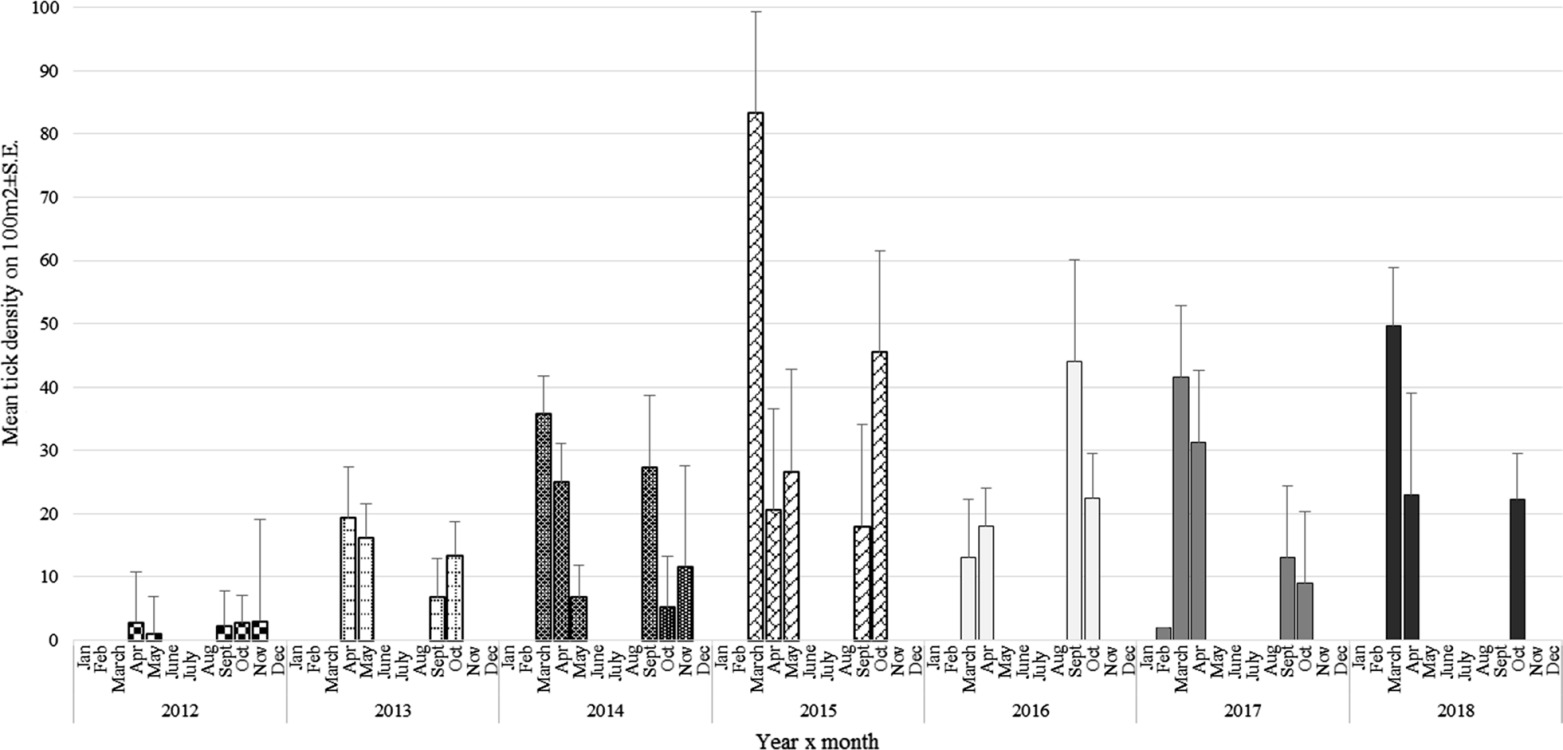
Mean tick abundance in endemic sites by year and month

Seasonal abundance of *D. reticulatus* in optimal habitats over a 7-year period is presented in Fig. 3. The highest annual mean density of *D. reticulatus* in endemic areas was observed in 2015 (38.82 ± 8.07) and the lowest in 2012 (2.03 ± 4.57) (main effect of YEAR on tick abundance: *F*_6, 135_ = 2.25, *P* = 0.02). The highest seasonal mean abundance was recorded in March (mean abundance = 42.27 ± 5.30) and April. In autumn, the highest density (about 18–19 ticks/100 m^2^) was observed in September and October (Fig. 3).

### Occurrence of canine babesiosis

#### Raw data collection

**Fig. 4:**
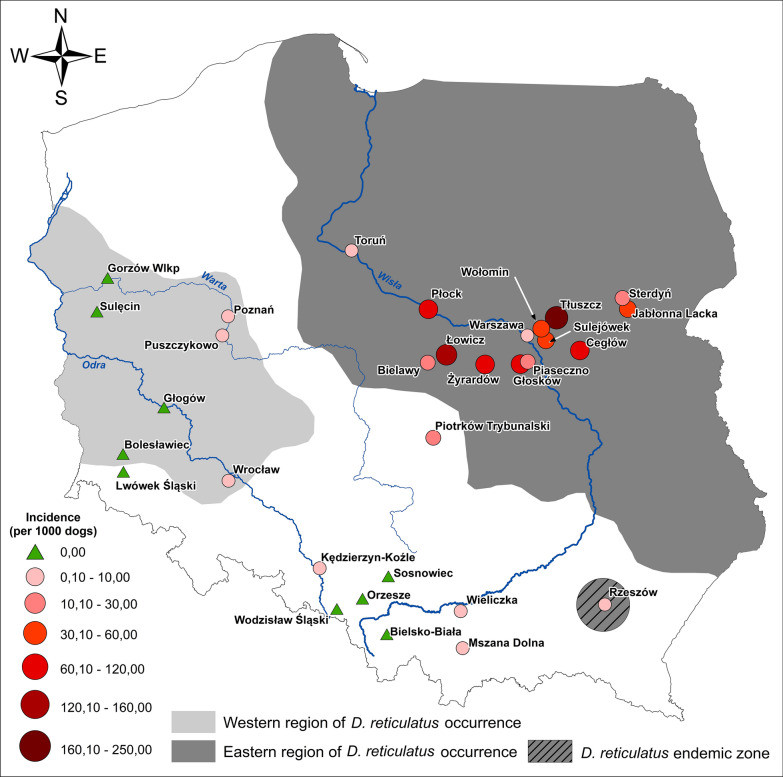
Incidence (per 1000 dogs) of canine babesiosis in veterinary clinics by current range of *D. reticulatus*

Data regarding canine babesiosis were collected from 47 clinics from three main regions of Poland (Fig. 4; Table 5), covering an estimated 79,204 dogs, including 11 small clinics (reporting 300–999 dogs annually), 20 clinics taking care of 1000–1500 dogs annually and 16 large clinics (1520–5500 dogs). The total number of babesiosis cases registered in 2018 by the VP was 1558, with the vast majority of cases (*n* = 1532) from Eastern and Central Poland (Eastern population/region of *D. reticulatus*) (Table 5). Only 19 cases of babesiosis were reported from Western Poland (Western population/region of *D. reticulatus*), and only seven cases were reported from clinics located in the gap zone, outside the current range of the tick *D. reticulatus* (Table 5).

**Table 5:** Veterinary clinics from three main regions (Western, Eastern and gap)

Region of *D. reticulatus* occurrence	Clinic	Longitude	Latitude	Total babesiosis cases	Total fatal cases	Number of dogs examined	Incidence (%)	Incidence/1000 dogs	Fatality/1000 dogs	Fatality (%) dogs
Eastern	Bielawy	52.075	19.659	14	0	500	2.80	28.00	0.00	0.00
	Cegłow	52.148	21.738	51	2	500	10.20	102.00	39.22	3.92
	Glosków	52.049	20.923	41	0	500	8.20	82.00	0.00	0.00
	Jabłonna Lacka	52.478	22.418	10	1	300	3.33	33.33	100.00	10.00
	Łowicz	52.139	19.917	197	10	1300	15.15	151.54	50.76	5.08
	Piaseczno 1	52.068	21.022	30	0	1000	3.00	30.00	0.00	0.00
	Piaseczno 2	52.060	20.983	97	1	4000	2.43	24.25	10.31	1.03
	Piotrków Trybunalski	51.444	19.725	22	0	1200	1.83	18.33	0.00	0.00
	Płock	52.524	19.676	300	55	3000	10.00	100.00	183.33	18.33
	Rzeszów	50.008	21.940	24	3	2400	1.00	10.00	125.00	12.50
	Sterdyń	52.572	22.353	15	2	500	3.00	30.00	133.33	13.33
	Sulejówek	52.242	21.280	38	0	1000	3.80	38.00	0.00	0.00
	Tłuszcz	52.429	21.435	361	10	1500	24.07	240.67	27.70	2.77
	Toruń 1	53.018	18.613	1	0	1500	0.07	0.67	0.00	0.00
	Toruń 2	53.018	18.613	7	0	500	1.40	14.00	0.00	0.00
	Toruń 3	53.018	18.613	19	0	1000	1.90	19.00	0.00	0.00
	Warszawa Bródno	52.288	21.028	3	0	500	0.60	6.00	0.00	0.00
	Warszawa Praga	52.239	21.086	21	0	500	4.20	42.00	0.00	0.00
	Warszawa Targówek	52.284	21.062	9	0	500	1.80	18.00	0.00	0.00
	Warszawa Wawer	52.223	21.141	16	1	2660	0.60	6.02	62.50	6.25
	Wołomin	52.342	21.221	33	2	1000	3.30	33.00	60.61	6.06
	Żyrardów 1	52.054	20.441	41	1	1521	2.70	26.96	24.39	2.44
	Żyrardów 2	52.065	20.441	30	1	500	6.00	60.00	33.33	3.33
	Żyrardów 3	52.065	20.441	152	4	1000	15.20	152.00	26.32	2.63
Total				1532	93	28,881	5.27	52.74	36.53	3.65
Gap	Bielsko-Biała	49.796	19.096	0	0	5500	0.00	0.00	0.00	0.00
	Kędzierzyn Koźle	50.347	18.216	3	0	1000	0.30	3.00	0.00	0.00
	Mszana dolna	49.673	20.079	3	0	1000	0.30	3.00	0.00	0.00
	Orzesze	50.094	18.780	0	0	500	0.00	0.00	0.00	0.00
	Sosnowiec	50.283	19.117	0	0	1000	0.00	0.00	0.00	0.00
	Wieliczka	49.984	20.060	1	0	3500	0.03	0.29	0.00	0.00
	Wodzisław Śląski	50.007	18.446	0	0	1200	0.00	0.00	0.00	0.00
Total				7	0	13,700	0.09	0.90	0.00	0.00
Western	Bolesławiec 1	51.260	15.566	0	0	2500	0.00	0.00	0.00	0.00
	Bolesławiec 2	51.260	15.566	0	0	1500	0.00	0.00	0.00	0.00
	Bolesławiec 3	51.260	15.566	0	0	1500	0.00	0.00	0.00	0.00
	Bolesławiec 4	51.260	15.566	0	0	1500	0.00	0.00	0.00	0.00
	Głogów	51.665	16.090	0	0	1500	0.00	0.00	0.00	0.00
	Gorzów Wlkp. 1	52.730	15.240	0	0	3000	0.00	0.00	0.00	0.00
	Gorzów Wlkp. 2	52.730	15.240	0	0	3000	0.00	0.00	0.00	0.00
	Lwówek śląski	51.111	15.583	0	0	2500	0.00	0.00	0.00	0.00
	Poznań 1	52.447	16.921	12	3	3400	0.35	3.53	250.00	25.00
	Poznań 2	52.447	16.921	1	0	2660	0.04	0.38	0.00	0.00
	Poznań 3	52.447	16.921	3	0	1500	0.00	0.00	0.00	0.00
	Puszczykowo	52.286	16.849	1	0	2300	0.04	0.43	0.00	0.00
	Sulęcin	52.443	15.117	0	0	1500	0.00	0.00	0.00	0.00
	Wrocław 1	51.068	16.989	2	0	1000	0.20	2.00	0.00	0.00
	Wrocław 2	51.133	17.025	0	0	3763	0.00	0.00	0.00	0.00
	Wrocław 3	51.133	17.025	0	0	3500	0.00	0.00	0.00	0.00
Total				19	3	36,623	0.04	0.40	15.63	1.56

### Annual incidence of babesiosis

The overall annual incidence of clinical babesiosis among the Polish dog population was 19.67/1000 dogs (1.97%), with marked differences among three regions of the country (Table 5). The annual incidence was low (less than 1 case per 1000 dogs) in Western Poland (new endemic region for ornate dog tick) and in an area historically free of this tick species. Incidence was 100 times as high (52.74/1000) among dogs from Eastern and Central Poland (Eastern tick population) (Table 5). In this region, incidence varied between 100 and 240 cases/1000 dogs in five clinics (10–24% of attending dogs); 24–82 cases/1000 dogs in 10 clinics and 0.7–19 cases/1000 dogs in eight clinics (Table 5). In the two remaining regions with overall low incidence, incidence varied only between 0 and 3.0 cases/1000 dogs (Table 5).

### Fatality in canine babesiosis

Fatalities comprised 2.40% of clinical cases, and similar fatality rates were recorded in Eastern and Central Poland (Table 5). Because of the low number of all cases in the two remaining regions, fatality rates varied between 0.00 and 25.00% (Table 5).

### Seasonal dynamics of clinical babesiosis

**Fig. 5:**
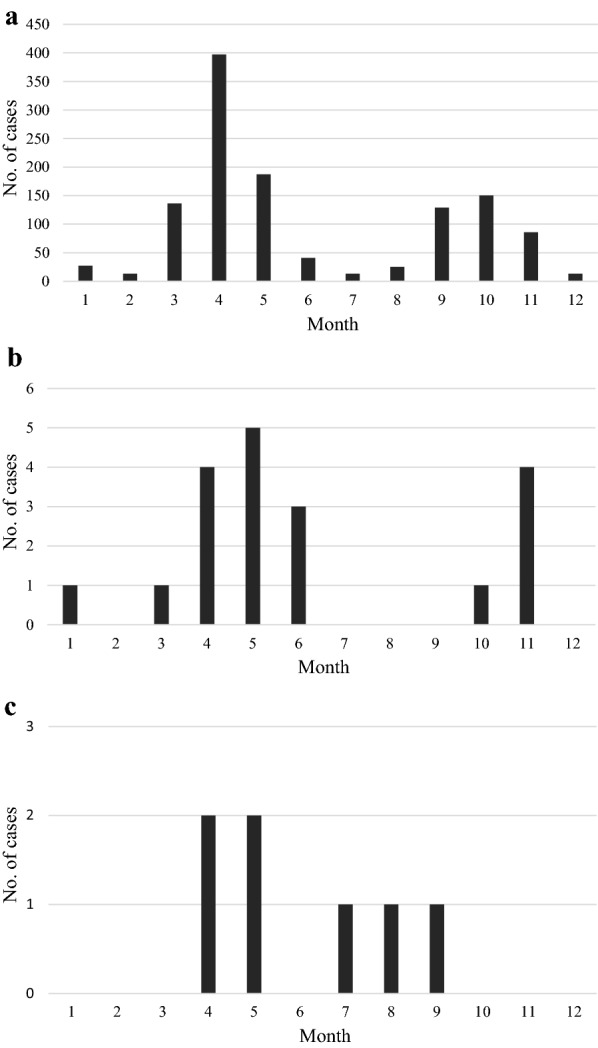
The number of babesiosis cases recorded in each month from **a** Eastern region, **b** Western region, **c** gap region

**Fig. 6:**
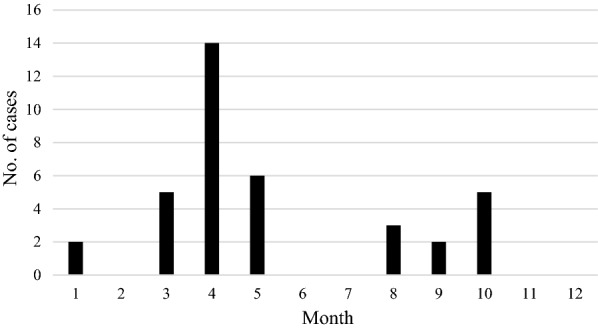
Number of total fatal cases of babesiosis per month

The total number of babesiosis cases recorded in each month in clinics from various regions of Poland is presented in Fig. 5. In all the clinics from the old endemic region (Eastern and Central Poland), a similar pattern of occurrence of babesiosis cases was recorded, clearly reflecting seasonal activity of *D. reticulatus* ticks (Fig. 5a). The number of cases increased from early to late spring, to peak in April–May, and a second (lower) peak was observed in October–November, following a reduction in cases in summer. A similar pattern was observed in the seasonal distribution of babesiosis cases from the new endemic region in Western Poland (Fig. 5b) but no clear pattern was found in the gap zone (Fig. 5c), probably reflecting erratic travelling of dogs/dog owners during the spring–summer period. In the old endemic region of *D. reticulatus*, clinical cases of babesiosis were recorded in all months across the year, including winter (Fig. 5a). Detailed data from each clinic is given in Additional file 2: Figure S1. Total number of fatal cases in the old endemic region followed the seasonal pattern of babesiosis occurrence, peaking in April and October 2018 (Fig. 6).

## Discussion

The present study revealed further expansion of each of the two Polish *D. reticulatus* populations between spring 2016 and autumn 2018, with marked colonization of the gap zone. For the first time, the seasonal shift in the borders of the range of ticks was estimated, suggesting continuing progress of expansion into the future. Data from veterinary clinics revealed a low risk of canine babesiosis in Western Poland and the gap zone, but a high incidence of infection and fatality in Central and Eastern Poland, in the endemic area of the Eastern tick population and its expansion zone. Based on our data, we were able to compile an updated map of the incidence of babesiosis in different regions of Poland, together with the current areas and limits of the occurrence of the tick *D. reticulatus.*

In comparison to our previous monitoring study in 2012–2014 [2], numerous new *D. reticulatus* foci were found during the period 2016–2018, with further colonization of the gap zone. Expansion was confirmed both by the detection of new *D. reticulatus*-positive sites in areas previously free of this tick species as confirmed in our earlier study, and by the dynamic changes in the borders of tick ranges (seasonal shift) and the distance separating the two tick populations. The calculation we conducted herein revealed that this tick species is continually expanding its range and is gradually but systematically colonizing the gap zone. The mean shift in the limits of the tick range was clearly higher for the Western population than the Eastern one. Thus, it seems very likely that the *D. reticulatus*-free area is going to disappear in 10–13 year’s time, and the two populations will then meet and merge. It would be of interest to compare this process with reports from other countries in Western, Central and Eastern Europe.

The reasons for the dynamic expansion of this tick species in the area of Central Europe, including Poland, are not understood. Generally, the spread of ticks into new areas is affected by a diverse range of biotic and abiotic factors [43], but knowledge of those factors specific to *D. reticulatus* is still limited. It has been shown that neither temperature nor duration of the growing seasons is a limiting factor for the spread of *D. reticulatus* in the territory of Poland [14]. One of the possible causes of tick spread to the new areas might be host mobility [44, 45], as tick hosts (especially medium-size or large mammals) obviously have a greater ability to migrate for longer distances than the ticks themselves. Increasing wildlife populations such as elk (*Alces alces*), roe deer (*Capreolus capreolus*) and red deer (*Cervus elaphus*) in Poland [46], all known to be good hosts for adult *D. reticulatus* [47–49], can facilitate colonization of the new areas.

In the present monitoring, a few *D. reticulatus*-positive sites appeared in one season and then reverted to negativity in the following seasons. Absence of ticks in the following season can be explained by the lack of appropriate habitats for maintenance of *D. reticulatus.* Occurrence of this tick species is strongly associated with the presence of open habitats (including deforestation), fragmentation of landscape within a large patch of homogeneous vegetation, and the presence of watercourses [14]. The most eastward and westward tick-positive sites for both the Western and Eastern populations were located in Greater Poland Voivodeship, which comprises highly agricultural areas (report from statistical office in Poznań, 2017). A single appearance of *D. reticulatus* in Sławoszewek (the most westward sites from the Eastern population) was noted at a site surrounded by cereal monocultures and located in close proximity to the KWB Konin SA brown coal opencast mine. It is possible that ticks were carried to this site by a wild animal, but without suitable habitat conditions, maintenance of a new focus of ticks was not possible. The temporal/ephemeral occurrence of ticks in several sites from both expansion zones is also supported by significantly lower densities/abundance of ticks in the expansion zones in comparison to endemic areas.

Interestingly, seasonal shifts in the borders of both tick population ranges were recorded during the present study along the river valleys, and this observation concurs with previous studies [2, 7, 12, 14, 24]. Thus, on the basis of current trends, we can predict with some confidence that eventually the two populations of *D. reticulatus* will meet in a river valley. The most likely is the Warta River valley—*D. reticulatus*-positive sites from both Eastern and Western populations were found there, creating one of the shortest distances between the two tick populations and facilitating expansion along a west–east axis (Fig. 1).

Comparison of the occurrence/distribution of both ticks and canine babesiosis clearly demonstrates that expansion of the tick range is accompanied by a concomitant expansion of this important tick-borne disease. Our study revealed a low risk of canine babesiosis in Western Poland and in the gap zone and a high incidence of disease and fatality in Central and Eastern Poland, in the endemic areas of the Eastern tick population and its expansion zone.

As we expected, on the basis of the prevalence *B. canis* in *D. reticulatus* ticks [23, 24], the highest number of babesiosis cases was reported in the region of endemicity of the Eastern *D. reticulatus* population. Here, incidence of babesiosis was up to 100 × higher in veterinary clinics compared to those located in the gap zone and Western regions. In another study [31], the number of babesiosis cases was six times higher in dogs in Eastern regions of Poland compared to the Western regions. Disparity in occurrence of canine babesiosis cannot be explained by the slightly lower abundance of ticks in Western Poland (present study), but is in accordance with differences in *B. canis* prevalence in these two tick populations. *Babesia canis* DNA has been detected only in Central and Eastern Poland (Eastern tick population) and was never detected in large samples of questing *D. reticulatus* collected in Western Poland throughout 2012–2018 (Dwużnik and Kiewra, unpublished data) [15, 27]. Seasonal occurrence of babesiosis in dogs from the Eastern region is strongly associated with a high tick density observed during the spring and autumn tick activity periods. Following Martinod and Gilot [5] and our earlier studies [21], we can define the Central and Eastern region of Poland as a hyperendemic region for canine babesiosis caused by *B. canis*.

Based on feedback from two clinics, one from the gap zone and one from Western region, *B. canis* infections were identified in dogs with a history of travel to the Eastern region of *D. reticulatus* occurrence. The dogs’ travel histories [50] to the *B. canis* high-risk region and the relatively low number of babesiosis cases in their home territories indicate that the disease was imported to the Western region rather than overlooked earlier locally. On the other hand, the emergence of a new tick-vectored pathogen in regions considered free of ticks may be the first sign of an expanding geographical coverage for this tick species [51]. However, single cases of babesiosis in the Western region suggest importation of infection from the hyperendemic Eastern region rather than established endemicity of babesiosis in the Western population of ticks. Even if *B. canis* has been established in the Western population, the very low numbers of recorded cases could reflect extremely low *B. canis* prevalence in ticks in this region. In a recent study in Bavaria, Germany (Western metapopulation), only one *B. canis*-positive tick (0.3%) was found among 301 questing *D. reticulatus* examined [52]. In contrast, among 60 ticks collected from vegetation in Western Ukraine (Eastern metapopulation), DNA of *B. canis* was identified in 12% of ticks [53].

An interesting pattern has been observed in Rzeszów city (at the border of the Carpathian mountain area), where a high incidence of *B. canis* infection in dogs together with the classical seasonal distribution of babesiosis cases suggest that a stable population of *D. reticulatus* occurs in this area. Moreover, new foci of *D. reticulatus* ticks have been observed even further south in the Przemyskie Foothills [54].

The information provided in this paper on the current range of *D. reticulatus* in Poland and the associated map (Fig. 1d) should be of interest in facilitating the prediction of infection risk with other pathogens vectored by this tick species, as for example *Rickettsia* spp. and tick-borne encephalitis virus [15, 55].

## Conclusions

In the present study we have determined the actual geographical range of the *D. reticulatus* tick in Poland and the seasonal/annual shift in limits to its range, and we have documented the continuing process of colonization of the gap zone. Moreover, we have documented the gradual, progressive disappearance of tick-free areas in Central Europe and predicted possible contact of the two tick metapopulations. We have also reported the emergence of canine babesiosis in the expansion zone of the Eastern *D. reticulatus* population and the much lower risk of babesiosis associated with the expansion of the Western tick population. Finally, the updated map of the distribution of *D. reticulatus* and babesiosis in different regions of Poland has allowed the prediction of the emergence of a range of other tick-borne diseases vectored by *D. reticulatus.*

## Supplementary Information

**Additional file 1**: **Table S1**. Number of collections during the monitoring study.

**Additional file 2**: **Figure S1**. Number of babesiosis cases recorded from veterinary clinics (Eastern, Western, gap region) per month.

## Data Availability

All data generated or analysed during this study are included in this published article and its additional information files.

## Abbreviations

SE: Southeastern
NE: Northeastern
VP: Veterinary practitioners
