# Supplementary material for: Monitoring the expansion of Dermacentor reticulatus and occurrence of canine babesiosis in Poland in 2016–2018
Source: Parasit Vectors. 2021 May 20;14:267. doi: 10.1186/s13071-021-04758-7 (PMC8138931; doi:10.1186/s13071-021-04758-7)
Supplement: Supplementary file 1 — Additional file 1: Table S1. Number of collections during the monitoring study. [file 13071_2021_4758_MOESM1_ESM.docx]

| **Year** | **Month** | **Season of tick collection** | **Voivodeship** | **Location** | **Region** | **Lattitude** | **Longitude** | **Mean densitty of tick/100m2** |
| --- | --- | --- | --- | --- | --- | --- | --- | --- |
| 2016 | 3 | Spring | Mazowieckie | Huta Partacka | Eastern region | 51.57047 | 20.24351 | 2.00 |
| 2016 | 3 | Spring | Mazowieckie | Karnice1 | Eastern region | 51.57025 | 20.25469 | 6.67 |
| 2016 | 3 | Spring | Mazowieckie | Karnice2 | Eastern region | 51.57120 | 20.26089 | 4.33 |
| 2016 | 3 | Spring | Mazowieckie | Karnice3 | Eastern region | 51.57077 | 20.26183 | 1.00 |
| 2016 | 3 | Spring | Mazowieckie | Korabiewice | Eastern region | 51.57156 | 20.26067 | 1.00 |
| 2016 | 3 | Spring | Mazowieckie | Korabiewice2 | Eastern region | 51.57061 | 20.24509 | 1.00 |
| 2016 | 4 | Spring | Łódzkie | Rawa Mazowiecka | Eastern expansion zone | 51.75020 | 20.22630 | 7.67 |
| 2016 | 4 | Spring | Łódzkie | Bełchów1 | Eastern expansion zone | 52.04000 | 20.05900 | 0.00 |
| 2016 | 4 | Spring | Łódzkie | Drzewica | Eastern expansion zone | 51.44330 | 20.47190 | 1.38 |
| 2016 | 4 | Spring | Łódzkie | Inowłódz | Eastern expansion zone | 51.53160 | 20.22590 | 4.88 |
| 2016 | 4 | Spring | Łódzkie | Józefina | Eastern expansion zone | 51.27760 | 18.70510 | 0.00 |
| 2016 | 4 | Spring | Łódzkie | Kałduny | Eastern expansion zone | 51.41440 | 19.37720 | 0.00 |
| 2016 | 4 | Spring | Łódzkie | Lubiszów Kolonia | Eastern expansion zone | 51.42360 | 20.28240 | 0.13 |
| 2016 | 4 | Spring | Łódzkie | Mzurki | Eastern expansion zone | 51.42750 | 19.48570 | 0.00 |
| 2016 | 4 | Spring | Łódzkie | Raków | Eastern expansion zone | 51.44460 | 19.72470 | 0.00 |
| 2016 | 4 | Spring | Łódzkie | Torzeniec | Eastern expansion zone | 51.36940 | 18.10790 | 0.00 |
| 2016 | 4 | Spring | Łódzkie | Wyszanów | Eastern expansion zone | 51.37090 | 18.16470 | 0.00 |
| 2016 | 4 | Spring | Łódzkie | Zagadki | Eastern expansion zone | 51.34460 | 19.12590 | 0.00 |
| 2016 | 4 | Spring | Świętokrzyskie | Trębowiec Krupów | Eastern expansion zone | 51.17530 | 21.07630 | 0.25 |
| 2016 | 4 | Spring | Mazowieckie | Adamowice (Powązki) | Eastern region | 51.94030 | 20.47410 | 6.67 |
| 2016 | 4 | Spring | Mazowieckie | Bolimów | Eastern region | 52.08176 | 20.17120 | 0.20 |
| 2016 | 4 | Spring | Mazowieckie | Budy Grabskie | Eastern region | 52.00220 | 20.21280 | 0.00 |
| 2016 | 4 | Spring | Mazowieckie | Doleck | Eastern region | 51.92180 | 20.29430 | 2.00 |
| 2016 | 4 | Spring | Mazowieckie | Drezno | Eastern region | 51.21080 | 21.61870 | 4.67 |
| 2016 | 4 | Spring | Mazowieckie | Gostynin | Eastern region | 52.39550 | 19.51830 | 22.40 |
| 2016 | 4 | Spring | Mazowieckie | Gulinek | Eastern region | 51.46220 | 21.00130 | 0.00 |
| 2016 | 4 | Spring | Mazowieckie | Jamki | Eastern region | 51.44210 | 20.71880 | 0.00 |
| 2016 | 4 | Spring | Mazowieckie | Kamion | Eastern region | 51.94610 | 20.25720 | 0.00 |
| 2016 | 4 | Spring | Mazowieckie | Kociołki | Eastern region | 51.54245 | 21.55774 | 3.67 |
| 2016 | 4 | Spring | Mazowieckie | Korabiewice | Eastern region | 51.95050 | 20.42810 | 4.00 |
| 2016 | 4 | Spring | Mazowieckie | Kury 1 | Eastern region | 52.41368 | 21.51203 | 0.50 |
| 2016 | 4 | Spring | Mazowieckie | Kury 2 | Eastern region | 52.41368 | 21.51203 | 6.50 |
| 2016 | 4 | Spring | Mazowieckie | Kury 3 | Eastern region | 52.41368 | 21.51203 | 13.93 |
| 2016 | 4 | Spring | Mazowieckie | Kury 4 | Eastern region | 52.41368 | 21.51203 | 5.00 |
| 2016 | 4 | Spring | Mazowieckie | Lipsko | Eastern region | 51.15750 | 21.63370 | 0.00 |
| 2016 | 4 | Spring | Mazowieckie | Owadów | Eastern region | 51.48825 | 21.17526 | 13.88 |
| 2016 | 4 | Spring | Mazowieckie | Pasztowa Wola | Eastern region | 51.14720 | 21.38210 | 3.20 |
| 2016 | 4 | Spring | Mazowieckie | Plecewice | Eastern region | 52.28498 | 20.27473 | 5.71 |
| 2016 | 4 | Spring | Mazowieckie | Pomarzanki | Eastern region | 52.37210 | 19.30330 | 0.00 |
| 2016 | 4 | Spring | Mazowieckie | Puszcza Mariańska | Eastern region | 51.97010 | 20.34610 | 4.17 |
| 2016 | 4 | Spring | Mazowieckie | Rawiczów | Eastern region | 51.92530 | 20.17790 | 0.10 |
| 2016 | 4 | Spring | Mazowieckie | Resztki | Eastern region | 52.38070 | 19.62860 | 2.67 |
| 2016 | 4 | Spring | Mazowieckie | Sady | Eastern region | 51.44670 | 20.67540 | 1.13 |
| 2016 | 4 | Spring | Mazowieckie | Seredzice | Eastern region | 51.16280 | 21.12680 | 0.00 |
| 2016 | 4 | Spring | Mazowieckie | Strobów | Eastern region | 51.91267 | 20.17192 | 0.00 |
| 2016 | 4 | Spring | Mazowieckie | Wierzbice | Eastern region | 51.27450 | 21.10530 | 0.00 |
| 2016 | 4 | Spring | Mazowieckie | Zakrzewska Wola | Eastern region | 51.45060 | 21.95690 | 14.00 |
| 2016 | 4 | Spring | Mazowieckie | Zofiówka | Eastern region | 51.15780 | 21.52370 | 0.00 |
| 2016 | 4 | Spring | Mazowieckie | Zwoleń | Eastern region | 51.33430 | 21.59020 | 4.13 |
| 2016 | 4 | Spring | Dolnośląskie | Kawice | Western region | 51.24400 | 16.42809 | 28.67 |
| 2016 | 4 | Spring | Dolnośląskie | Pogorzeliska | Western region | 51.46430 | 15.95100 | 18.67 |
| 2016 | 4 | Spring | Wielkopolskie | Kuźnica Zbąska | Western region | 52.23180 | 16.10573 | 24.50 |
| 2016 | 4 | Spring | Dolnośląskie | Wiszna Mała 1 | Western expansion zone | 51.24486 | 17.03178 | 0.80 |
| 2016 | 4 | Spring | Dolnośląskie | Wiszna Mała 2 | Western expansion zone | 51.23710 | 17.05790 | 6.80 |
| 2016 | 4 | Spring | Lubuskie | Policko | Western expansion zone | 52.45870 | 15.70360 | 1.50 |
| 2016 | 4 | Spring | Lubuskie | Rokitno | Western expansion zone | 52.53390 | 15.65360 | 2.73 |
| 2016 | 4 | Spring | Lubuskie | Skwierzyna | Western expansion zone | 52.61080 | 15.52440 | 0.33 |
| 2016 | 4 | Spring | Lubuskie | Świechocin | Western expansion zone | 52.49670 | 15.89720 | 0.15 |
| 2016 | 4 | Spring | Wielkopolskie | Borówiec | Western expansion zone | 52.28180 | 17.04316 | 0.00 |
| 2016 | 4 | Spring | Wielkopolskie | Glinka | Western expansion zone | 51.70440 | 16.52150 | 0.00 |
| 2016 | 4 | Spring | Wielkopolskie | Góra | Western expansion zone | 51.66710 | 16.57030 | 0.00 |
| 2016 | 4 | Spring | Wielkopolskie | Grabce | Western expansion zone | 51.51980 | 16.86770 | 1.00 |
| 2016 | 4 | Spring | Wielkopolskie | Grubsko | Western expansion zone | 52.42870 | 16.11450 | 0.00 |
| 2016 | 4 | Spring | Wielkopolskie | Konin | Western expansion zone | 52.47340 | 16.22720 | 0.50 |
| 2016 | 4 | Spring | Wielkopolskie | Linie | Western expansion zone | 52.48930 | 16.13840 | 0.00 |
| 2016 | 4 | Spring | Wielkopolskie | Łowyń | Western expansion zone | 52.50100 | 15.92250 | 0.00 |
| 2016 | 4 | Spring | Wielkopolskie | Mikstat | Western expansion zone | 51.52540 | 17.99080 | 0.00 |
| 2016 | 4 | Spring | Wielkopolskie | Mosina | Western expansion zone | 52.24300 | 16.84030 | 0.00 |
| 2016 | 4 | Spring | Wielkopolskie | Nowy Tomyśl | Western expansion zone | 52.32790 | 16.11530 | 16.80 |
| 2016 | 4 | Spring | Wielkopolskie | Obra | Western expansion zone | 52.06911 | 16.04106 | 2.88 |
| 2016 | 4 | Spring | Wielkopolskie | Odolanów | Western expansion zone | 51.57530 | 17.82900 | 1.88 |
| 2016 | 4 | Spring | Wielkopolskie | Trzebosz | Western expansion zone | 51.67280 | 16.73180 | 0.00 |
| 2016 | 4 | Spring | Wielkopolskie | Rogalin | Western expansion zone | 52.21992 | 16.92339 | 0.00 |
| 2016 | 4 | Spring | Wielkopolskie | Trzebosz | Western expansion zone | 51.66800 | 16.73150 | 0.00 |
| 2016 | 4 | Spring | Dolnośląskie | Henrykowice | Western expansion zone | 51.51900 | 17.45290 | 0.00 |
| 2016 | 4 | Spring | Dolnośląskie | Kobylice | Western expansion zone | 51.34070 | 17.07220 | 0.00 |
| 2016 | 4 | Spring | Dolnośląskie | Koniowo | Western expansion zone | 51.44410 | 17.06400 | 0.00 |
| 2016 | 4 | Spring | Dolnośląskie | Milicz Słaboszowice | Western expansion zone | 51.52550 | 17.30100 | 0.00 |
| 2016 | 4 | Spring | Dolnośląskie | Osiek | Western expansion zone | 51.46570 | 17.00980 | 0.00 |
| 2016 | 4 | Spring | Dolnośląskie | Ruda Żmigrodzka | Western expansion zone | 51.50380 | 16.96220 | 0.60 |
| 2016 | 4 | Spring | Dolnośląskie | Szczytkowice | Western expansion zone | 51.36350 | 17.08580 | 0.00 |
| 2016 | 9 | Autumn | Warmińsko-Mazurskie | Urwitałt | Eastern region | 53.80932 | 21.64764 | 44.00 |
| 2016 | 10 | Autumn | Łódzkie | Trzcianna( Suliszynek) | Eastern expansion zone | 51.93497 | 20.27649 | 0.00 |
| 2016 | 10 | Autumn | Mazowieckie | Nowy Kurzeszyn | Eastern expansion zone | 51.82577 | 20.27326 | 1.50 |
| 2016 | 10 | Autumn | Łódzkie | Rawa Mazowiecka | Eastern expansion zone | 51.77977 | 20.25233 | 0.00 |
| 2016 | 10 | Autumn | Mazowieckie | Korabiewice | Eastern region | 51.95094 | 20.42969 | 5.33 |
| 2016 | 10 | Autumn | Mazowieckie | Podkońska Wola | Eastern region | 51.71560 | 20.20628 | 0.00 |
| 2016 | 10 | Autumn | Mazowieckie | Puszcza Mariańska | Eastern region | 51.97026 | 20.34676 | 0.60 |
| 2016 | 10 | Autumn | Mazowieckie | Stare Budy | Eastern region | 52.07974 | 20.48895 | 3.33 |
| 2016 | 10 | Autumn | Mazowieckie | Stoski 1 | Eastern region | 52.40923 | 21.50913 | 1.33 |
| 2016 | 10 | Autumn | Mazowieckie | Stoski 2 | Eastern region | 52.40923 | 21.50913 | 6.00 |
| 2016 | 10 | Autumn | Mazowieckie | Stoski 3 | Eastern region | 52.40923 | 21.50913 | 50.00 |
| 2016 | 10 | Autumn | Lubuskie | Popowice | Western region | 51.75545 | 15.25183 | 4.40 |
| 2016 | 10 | Autumn | Dolnośląskie | Cesarzowice | Western region | 51.11182 | 16.56447 | 7.40 |
| 2016 | 10 | Autumn | Dolnośląskie | Biedaszków Mały | Western expansion zone | 51.39966 | 17.09868 | 5.20 |
| 2016 | 10 | Autumn | Lubuskie | Baczyna | Western expansion zone | 52.74998 | 15.13779 | 1.00 |
| 2016 | 10 | Autumn | Lubuskie | Murzynowo | Western expansion zone | 52.65045 | 15.46205 | 0.00 |
| 2016 | 10 | Autumn | Lubuskie | Sciechów | Western expansion zone | 52.81714 | 14.98125 | 0.20 |
| 2016 | 10 | Autumn | Lubuskie | Zamyślin | Western expansion zone | 52.64595 | 15.78013 | 0.20 |
| 2016 | 10 | Autumn | Wielkopolskie | Bonikowo | Western expansion zone | 52.11619 | 16.63196 | 0.40 |
| 2016 | 10 | Autumn | Wielkopolskie | Bystrzek | Western expansion zone | 52.088206. | 17.08981 | 0.40 |
| 2016 | 10 | Autumn | Wielkopolskie | Grodzisk Wielkopolski | Western expansion zone | 52.23941 | 16.34532 | 0.40 |
| 2016 | 10 | Autumn | Wielkopolskie | Kuślin | Western expansion zone | 52.36124 | 16.29707 | 0.20 |
| 2016 | 10 | Autumn | Wielkopolskie | Lewiczynek | Western expansion zone | 52.43905 | 15.95955 | 0.20 |
| 2016 | 10 | Autumn | Wielkopolskie | Miejska Górka | Western expansion zone | 51.65156 | 16.92824 | 0.00 |
| 2016 | 10 | Autumn | Wielkopolskie | Muchocin | Western expansion zone | 52.59837 | 15.85723 | 0.00 |
| 2016 | 10 | Autumn | Wielkopolskie | Olsza | Western expansion zone | 51.54670 | 17.06737 | 0.00 |
| 2016 | 10 | Autumn | Wielkopolskie | Piaski | Western expansion zone | 51.88335 | 17.08261 | 0.00 |
| 2016 | 10 | Autumn | Wielkopolskie | Pogorzelica | Western expansion zone | 52.13971 | 17.58873 | 0.00 |
| 2016 | 10 | Autumn | Wielkopolskie | Wytomyśl | Western expansion zone | 52.38700 | 16.17535 | 0.60 |
| 2016 | 10 | Autumn | Zachodniopomorskie | Czerna | Western expansion zone | 51.52963 | 15.23219 | 0.00 |
| 2016 | 10 | Autumn | Zachodniopomorskie | Myślibórz | Western expansion zone | 52.94152 | 14.87199 | 0.00 |
| 2016 | 10 | Autumn | Zachodniopomorskie | Otanów | Western expansion zone | 52.99446 | 14.83788 | 0.00 |
| 2016 | 10 | Autumn | Zachodniopomorskie | Pełczyce | Western expansion zone | 50.96670 | 16.95563 | 0.00 |
| 2016 | 10 | Autumn | Zachodniopomorskie | Polesiny | Western expansion zone | 53.04887 | 14.48390 | 0.00 |
| 2016 | 10 | Autumn | Dolnośląskie | Groblice | Western expansion zone | 51.01623 | 17.17816 | 0.00 |
| 2016 | 10 | Autumn | Dolnośląskie | Skałka | Western expansion zone | 51.09688 | 16.83003 | 6.40 |
| 2016 | 10 | Autumn | Dolnośląskie | Szymanów | Western expansion zone | 51.20022 | 17.00000 | 5.80 |
| 2016 | 10 | Autumn | Dolnośląskie | Wrocław | Western expansion zone | 51.15737 | 16.97586 | 5.00 |
| 2016 | 10 | Autumn | Dolnośląskie | Żórawina | Western expansion zone | 50.98690 | 17.01271 | 0.00 |
| 2016 | 10 | Autumn | Dolnośląskie | Kąty Wrocławskie | Western expansion zone | 51.01631 | 16.75501 | 0.40 |
| 2016 | 10 | Autumn | Dolnośląskie | Kobylice | Western expansion zone | 51.33699 | 17.07000 | 0.00 |
| 2016 | 10 | Autumn | Dolnośląskie | Sułów | Western expansion zone | 51.49328 | 17.15214 | 0.40 |
| 2017 | 3 | Spring | Łódzkie | Bełchów2 | Eastern expansion zone | 52.01371 | 20.02561 | 3.83 |
| 2017 | 3 | Spring | Łódzkie | Borysław | Eastern expansion zone | 51.84629 | 20.08253 | 0.00 |
| 2017 | 3 | Spring | Łódzkie | Budziszyce( Nowy Józefów) | Eastern expansion zone | 51.67937 | 19.94729 | 0.00 |
| 2017 | 3 | Spring | Łódzkie | Dzierżgów | Eastern expansion zone | 52.04216 | 20.03625 | 0.88 |
| 2017 | 3 | Spring | Łódzkie | Inowłódz | Eastern expansion zone | 51.31894 | 20. 13554 | 0.00 |
| 2017 | 3 | Spring | Łódzkie | Jeziorki | Eastern expansion zone | 52.20410 | 20.05427 | 0.50 |
| 2017 | 3 | Spring | Łódzkie | Jeżów | Eastern expansion zone | 51.82032 | 19.96727 | 0.00 |
| 2017 | 3 | Spring | Łódzkie | Łyszkowice | Eastern expansion zone | 51.98622 | 19.91374 | 0.00 |
| 2017 | 3 | Spring | Łódzkie | Maków | Eastern expansion zone | 51.93578 | 20.07025 | 0.00 |
| 2017 | 3 | Spring | Łódzkie | Miedzna Drewniana | Eastern expansion zone | 51.32375 | 20.26925 | 0.00 |
| 2017 | 3 | Spring | Łódzkie | Petrykozy | Eastern expansion zone | 51.29853 | 20.34228 | 0.00 |
| 2017 | 3 | Spring | Łódzkie | Petrynów | Eastern expansion zone | 51.70473 | 20.09357 | 0.22 |
| 2017 | 3 | Spring | Łódzkie | Podkońksa Wola | Eastern expansion zone | 51.17607 | 20.20582 | 0.67 |
| 2017 | 3 | Spring | Łódzkie | Rękawiec | Eastern expansion zone | 51.67541 | 20.00971 | 0.00 |
| 2017 | 3 | Spring | Łódzkie | Strzelczew | Eastern expansion zone | 52.13798 | 19.98478 | 0.63 |
| 2017 | 3 | Spring | Świętokrzyskie | Rogówek | Eastern expansion zone | 51.26283 | 20.47715 | 0.00 |
| 2017 | 3 | Spring | Mazowieckie | Alojzów | Eastern region | 51.22848 | 21.24564 | 0.00 |
| 2017 | 3 | Spring | Mazowieckie | Białobrzegi | Eastern region | 51.66445 | 20.94700 | 6.40 |
| 2017 | 3 | Spring | Mazowieckie | Budy Zosine | Eastern region | 52.09349 | 20.51521 | 4.43 |
| 2017 | 3 | Spring | Mazowieckie | Dębowa Góra | Eastern region | 51.90550 | 20.12974 | 1.40 |
| 2017 | 3 | Spring | Mazowieckie | Golędzin | Eastern region | 51.41240 | 20.97299 | 3.00 |
| 2017 | 3 | Spring | Mazowieckie | Jaszowice | Eastern region | 51.41988 | 20.94812 | 9.17 |
| 2017 | 3 | Spring | Mazowieckie | Kociołki | Eastern region | 51.54245 | 21.55774 | 2.13 |
| 2017 | 3 | Spring | Mazowieckie | Kowala | Eastern region | 51.32423 | 21.07455 | 30.50 |
| 2017 | 3 | Spring | Mazowieckie | Krakowa Góra | Eastern region | 51.32158 | 20.66409 | 0.50 |
| 2017 | 3 | Spring | Mazowieckie | Lipsko | Eastern region | 51.14760 | 21.64316 | 0.00 |
| 2017 | 3 | Spring | Mazowieckie | Maruszów | Eastern region | 51.08632 | 21.61987 | 0.00 |
| 2017 | 3 | Spring | Mazowieckie | Owadów | Eastern region | 51.48825 | 21.17526 | 32.80 |
| 2017 | 3 | Spring | Mazowieckie | Pasztowa Wola | Eastern region | 51.08832 | 21.22927 | 2.83 |
| 2017 | 3 | Spring | Mazowieckie | Plecewice | Eastern region | 52.28498 | 20.27473 | 6.38 |
| 2017 | 3 | Spring | Mazowieckie | Podsuliszka | Eastern region | 51.24096 | 21.18053 | 5.00 |
| 2017 | 3 | Spring | Mazowieckie | Ryków | Eastern region | 51.31727 | 20.76844 | 0.75 |
| 2017 | 3 | Spring | Mazowieckie | Sienna | Eastern region | 51.08296 | 21.46009 | 0.00 |
| 2017 | 3 | Spring | Mazowieckie | Stoski | Eastern region | 52.40923 | 21.50913 | 50.50 |
| 2017 | 3 | Spring | Mazowieckie | Szydłowiec | Eastern region | 51.23046 | 20.86955 | 0.13 |
| 2017 | 3 | Spring | Mazowieckie | Wieniawa | Eastern region | 51.34750 | 20.79506 | 1.60 |
| 2017 | 3 | Spring | Mazowieckie | Zwoleń | Eastern region | 51.33543 | 21.59029 | 6.33 |
| 2017 | 3 | Spring | Mazowieckie | Żdarów | Eastern region | 52.23808 | 20.16233 | 12.20 |
| 2017 | 3 | Spring | Lubuskie | Trzciel/Rybojady | Western region | 52.39710 | 15.84251 | 5.50 |
| 2017 | 3 | Spring | Lubuskie | Grotów | Western expansion zone | 52.74228 | 15.85934 | 0.00 |
| 2017 | 3 | Spring | Wielkopolskie | Biała | Western expansion zone | 52.83954 | 16.34469 | 0.00 |
| 2017 | 3 | Spring | Wielkopolskie | Ciszkowo | Western expansion zone | 52.87875 | 16.45168 | 0.00 |
| 2017 | 3 | Spring | Wielkopolskie | Gorzyń | Western expansion zone | 52.56414 | 15.89750 | 0.00 |
| 2017 | 3 | Spring | Wielkopolskie | Kuślin | Western expansion zone | 52.36124 | 16.29707 | 1.33 |
| 2017 | 3 | Spring | Wielkopolskie | Łowyń | Western expansion zone | 52.49977 | 15.91678 | 0.00 |
| 2017 | 3 | Spring | Wielkopolskie | Mściszewo | Western expansion zone | 52.58018 | 16.98597 | 0.00 |
| 2017 | 3 | Spring | Wielkopolskie | Nowe Kniejce | Western expansion zone | 52.75020 | 15.99983 | 0.00 |
| 2017 | 3 | Spring | Wielkopolskie | Sępolno | Western expansion zone | 52.37593 | 16.03074 | 3.60 |
| 2017 | 3 | Spring | Wielkopolskie | Słonawy | Western expansion zone | 52.66543 | 16.75021 | 0.00 |
| 2017 | 3 | Spring | Wielkopolskie | Stobnicko | Western expansion zone | 52.71282 | 16.55169 | 0.00 |
| 2017 | 3 | Spring | Wielkopolskie | Wacławów | Western expansion zone | 52.23321 | 17.98901 | 0.00 |
| 2017 | 4 | Spring | Kujawsko-pomorskie | Barcin | Eastern expansion zone | 52.85078 | 17.96029 | 0.00 |
| 2017 | 4 | Spring | Łódzkie | Dąbkowice | Eastern expansion zone | 52.06508 | 19.87396 | 1.3 |
| 2017 | 4 | Spring | Wielkopolskie | Katarzyna | Eastern expansion zone | 52.31615 | 18.88637 | 0.5 |
| 2017 | 4 | Spring | Wielkopolskie | Sójki | Eastern expansion zone | 52.28031 | 19.37378 | 0.8 |
| 2017 | 4 | Spring | Mazowieckie | Adamowice | Eastern region | 51.93475 | 20.47222 | 24.00 |
| 2017 | 4 | Spring | Mazowieckie | Arciechów | Eastern region | 52.49580 | 21.11566 | 9.33 |
| 2017 | 4 | Spring | Mazowieckie | Gole | Eastern region | 52.40619 | 19.09193 | 1.00 |
| 2017 | 4 | Spring | Mazowieckie | Korabiewice | Eastern region | 51.95094 | 20.42969 | 43.00 |
| 2017 | 4 | Spring | Mazowieckie | Siekierki | Eastern region | 52.20404 | 21.08427 | 19.50 |
| 2017 | 4 | Spring | Dolnośląskie | Kawice | Western region | 51.22575 | 16.44067 | 2.33 |
| 2017 | 4 | Spring | Lubuskie | Krepa | Western region | 52.01080 | 15.54233 | 12.00 |
| 2017 | 4 | Spring | Lubuskie | Popowice | Western region | 51.75545 | 15.25183 | 2.20 |
| 2017 | 4 | Spring | Lubuskie | Przemków | Western region | 51.53418 | 15.77835 | 0.00 |
| 2017 | 4 | Spring | Lubuskie | Trzciel | Western region | 52.34482 | 15.87576 | 3.75 |
| 2017 | 4 | Spring | Wielkopolskie | Kuźnica Zbąska | Western region | 52.23260 | 16.12102 | 3.6 |
| 2017 | 4 | Spring | Wielkopolskie | Zakrzewko | Western region | 52.21492 | 15.97917 | 0.00 |
| 2017 | 4 | Spring | Dolnośląskie | Chróstnik | Western region | 51.36125 | 16.15577 | 3.60 |
| 2017 | 4 | Spring | Dolnośląskie | Brzeg Dolny | Western expansion zone | 51.26023 | 16.69594 | 3.00 |
| 2017 | 4 | Spring | Lubuskie | Janczewo | Western expansion zone | 52.76611 | 15.34509 | 1.17 |
| 2017 | 4 | Spring | Lubuskie | Lipki Wielkie | Western expansion zone | 52.72309 | 15.50205 | 0.00 |
| 2017 | 4 | Spring | Lubuskie | Rózanki | Western expansion zone | 52.79521 | 15.29428 | 0.00 |
| 2017 | 4 | Spring | Lubuskie | Skrzynica | Western expansion zone | 52.63111 | 15.67709 | 0.00 |
| 2017 | 4 | Spring | Lubuskie | Skwierzyna | Western expansion zone | 52.60936 | 15.48446 | 0.00 |
| 2017 | 4 | Spring | Wielkopolskie | Bonikowo | Western expansion zone | 52.11619 | 16.63196 | 0.0 |
| 2017 | 4 | Spring | Wielkopolskie | Boruja | Western expansion zone | 52.23005 | 16.08014 | 0.2 |
| 2017 | 4 | Spring | Wielkopolskie | Kowaliki | Western expansion zone | 51.60618 | 16.91093 | 0.40 |
| 2017 | 4 | Spring | Wielkopolskie | Narty | Western expansion zone | 52.38472 | 19.18620 | 0.00 |
| 2017 | 4 | Spring | Wielkopolskie | Niedrzew | Western expansion zone | 52.32914 | 19.36329 | 2.3 |
| 2017 | 4 | Spring | Wielkopolskie | Nowiny Brdowskie | Western expansion zone | 52.33310 | 18.73269 | 0.00 |
| 2017 | 4 | Spring | Wielkopolskie | Pogorzelica | Western expansion zone | 52.13962 | 17.58845 | 0.0 |
| 2017 | 4 | Spring | Wielkopolskie | Sobota | Western expansion zone | 52.66564 | 16.75029 | 1.3 |
| 2017 | 4 | Spring | Wielkopolskie | Śrem | Western expansion zone | 52.08752 | 16.98591 | 0.2 |
| 2017 | 4 | Spring | Wielkopolskie | Zamyślin | Western expansion zone | 52.64595 | 15.78013 | 0.00 |
| 2017 | 4 | Spring | Zachodniopomorskie | Banie | Western expansion zone | 53.09750 | 14.65297 | 0.3 |
| 2017 | 4 | Spring | Zachodniopomorskie | Dalszewo | Western expansion zone | 53.30112 | 14.52547 | 0.0 |
| 2017 | 4 | Spring | Zachodniopomorskie | Gajki | Western expansion zone | 53.20120 | 14.50354 | 0.0 |
| 2017 | 4 | Spring | Zachodniopomorskie | Kozielice | Western expansion zone | 53.12777 | 14.85161 | 0.0 |
| 2017 | 4 | Spring | Zachodniopomorskie | Lipiany | Western expansion zone | 53.01543 | 14.97059 | 0.0 |
| 2017 | 4 | Spring | Zachodniopomorskie | Moczowo | Western expansion zone | 52.97300 | 15.18926 | 0.0 |
| 2017 | 4 | Spring | Dolnośląskie | Budycze | Western expansion zone | 51.32934 | 17.18473 | 0.00 |
| 2017 | 4 | Spring | Dolnośląskie | Jakubowo | Western expansion zone | 51.43076 | 15.88398 | 0.67 |
| 2017 | 4 | Spring | Dolnośląskie | Lasowice | Western expansion zone | 51.45435 | 17.26813 | 0.00 |
| 2017 | 4 | Spring | Dolnośląskie | Piersno | Western expansion zone | 51.28160 | 17.15774 | 0.00 |
| 2017 | 4 | Spring | Dolnośląskie | Stara Góra | Western expansion zone | 51.64866 | 16.51296 | 1.00 |
| 2017 | 4 | Spring | Dolnośląskie | Stary Wołów | Western expansion zone | 51.35927 | 16.63671 | 0.24 |
| 2017 | 4 | Spring | Dolnośląskie | Sułów | Western expansion zone | 51.49328 | 17.15214 | 0.00 |
| 2017 | 4 | Spring | Dolnośląskie | Śleszów | Western expansion zone | 51.54230 | 16.53644 | 7.75 |
| 2017 | 4 | Spring | Dolnośląskie | Świętoszyn | Western expansion zone | 51.53062 | 17.24543 | 0.00 |
| 2017 | 4 | Spring | Dolnośląskie | Wiewierz | Western expansion zone | 51.62492 | 16.71991 | 0.25 |
| 2017 | 9 | Autumn | Kujawsko-Pomorskie | Biale Błota | Eastern expansion zone | 52.89722 | 18.69341 | 0.0 |
| 2017 | 9 | Autumn | Kujawsko-Pomorskie | Cetty | Eastern expansion zone | 52.38489 | 18.96138 | 0.2 |
| 2017 | 9 | Autumn | Kujawsko-Pomorskie | Koronowo | Eastern expansion zone | 53.31464 | 17.96716 | 0.13 |
| 2017 | 9 | Autumn | Kujawsko-Pomorskie | Kruszwica | Eastern expansion zone | 52.68535 | 18.32708 | 0.0 |
| 2017 | 9 | Autumn | Kujawsko-Pomorskie | Modzerowo | Eastern expansion zone | 52.34435 | 18.75770 | 0.0 |
| 2017 | 9 | Autumn | Kujawsko-Pomorskie | Nowa Wioska | Eastern expansion zone | 52.95795 | 18.13315 | 0.0 |
| 2017 | 9 | Autumn | Kujawsko-Pomorskie | Osielsk | Eastern expansion zone | 53.20956 | 18.05884 | 2.9 |
| 2017 | 9 | Autumn | Kujawsko-Pomorskie | Piła | Eastern expansion zone | 53.51395 | 17.88431 | 0.0 |
| 2017 | 9 | Autumn | Kujawsko-Pomorskie | Świecie | Eastern expansion zone | 53.41491 | 18.45496 | 0.0 |
| 2017 | 9 | Autumn | Kujawsko-Pomorskie | Wąwóz Goły Jon | Eastern expansion zone | 53.63972 | 18.15438 | 0.0 |
| 2017 | 9 | Autumn | Kujawsko-Pomorskie | Wełpin | Eastern expansion zone | 53.52217 | 18.00826 | 0.0 |
| 2017 | 9 | Autumn | Kujawsko-Pomorskie | Wieniec | Eastern expansion zone | 52.65562 | 18.94277 | 0.0 |
| 2017 | 9 | Autumn | Kujawsko-Pomorskie | Wieniec 2 | Eastern expansion zone | 52.64530 | 18.93063 | 0.0 |
| 2017 | 9 | Autumn | Kujawsko-Pomorskie | Żur | Eastern expansion zone | 53.55727 | 18.36242 | 0.0 |
| 2017 | 9 | Autumn | Łódzkie | Bełchów 2 | Eastern expansion zone | 52.01371 | 20.02561 | 1.13 |
| 2017 | 9 | Autumn | Łódzkie | Domaniewice | Eastern expansion zone | 52.03562 | 19.85951 | 2.60 |
| 2017 | 9 | Autumn | Łódzkie | Maków | Eastern expansion zone | 51.93803 | 20.06783 | 0.00 |
| 2017 | 9 | Autumn | Łódzkie | Oszkowice | Eastern expansion zone | 52.08343 | 19.56167 | 0.10 |
| 2017 | 9 | Autumn | Łódzkie | Sobota | Eastern expansion zone | 52.11127 | 19.68434 | 0.80 |
| 2017 | 9 | Autumn | Łódzkie | Strzyboga | Eastern expansion zone | 51.91628 | 20.18895 | 0.17 |
| 2017 | 9 | Autumn | Mazowieckie | Osiny | Eastern expansion zone | 52.35656 | 19.37981 | 0.38 |
| 2017 | 9 | Autumn | Wielkopolskie | Ośno Górne | Eastern expansion zone | 52.38349 | 18.56762 | 0.00 |
| 2017 | 9 | Autumn | Wielkopolskie | Wymysłowo | Eastern expansion zone | 52.39507 | 18.54241 | 0.00 |
| 2017 | 9 | Autumn | Mazowieckie | Kaczkowizna | Eastern region | 52.26279 | 19.73394 | 0.30 |
| 2017 | 9 | Autumn | Mazowieckie | Korabiewice | Eastern region | 51.95050 | 20.42810 | 4.00 |
| 2017 | 9 | Autumn | Mazowieckie | Plecewice | Eastern region | 52.28593 | 20.27635 | 3.92 |
| 2017 | 9 | Autumn | Mazowieckie | Stoski | Eastern region | 52.40941 | 21.50862 | 21.50 |
| 2017 | 10 | Autumn | Świętokrzyskie | Skarżysko Kamienna | Eastern expansion zone | 51.14708 | 20.87178 | 0.00 |
| 2017 | 10 | Autumn | Mazowieckie | Adamowice | Eastern region | 51.93475 | 20.47222 | 3.25 |
| 2017 | 10 | Autumn | Mazowieckie | Białobrzegi | Eastern region | 51.66445 | 20.94700 | 5.83 |
| 2017 | 10 | Autumn | Mazowieckie | Gulinek | Eastern region | 51.46388 | 21.00221 | 4.67 |
| 2017 | 10 | Autumn | Mazowieckie | Jaszkowice | Eastern region | 51.42047 | 20.96341 | 2.86 |
| 2017 | 10 | Autumn | Mazowieckie | Kamion | Eastern region | 51.94610 | 20.25720 | 0.00 |
| 2017 | 10 | Autumn | Mazowieckie | Krakowa Góra | Eastern region | 51.32158 | 20.66409 | 2.00 |
| 2017 | 10 | Autumn | Mazowieckie | Owadów | Eastern region | 51.48825 | 21.17526 | 13.50 |
| 2017 | 10 | Autumn | Mazowieckie | Puszcza Mariańska | Eastern region | 51.97010 | 20.34610 | 0.17 |
| 2017 | 10 | Autumn | Mazowieckie | Ryków | Eastern region | 51.32706 | 20.78070 | 3.33 |
| 2017 | 10 | Autumn | Mazowieckie | Stanisławów | Eastern region | 51.24609 | 20.78781 | 1.40 |
| 2017 | 10 | Autumn | Mazowieckie | Szydłowiec | Eastern region | 51.23046 | 20.86955 | 6.33 |
| 2017 | 10 | Autumn | Mazowieckie | Wieniawa | Eastern region | 51.34750 | 20.79506 | 4.00 |
| 2017 | 10 | Autumn | Mazowieckie | Żyrardów | Eastern region | 52.07438 | 20.46676 | 4.20 |
| 2017 | 10 | Autumn | Lubuskie | Bytom Odrzański | Western region | 51.73296 | 15.80051 | 4.00 |
| 2017 | 10 | Autumn | Lubuskie | Piaski | Western region | 51.85476 | 15.35352 | 6.75 |
| 2017 | 10 | Autumn | Lubuskie | Trzciel | Western region | 52.37187 | 15.86673 | 23.00 |
| 2017 | 10 | Autumn | Dolnośląskie | Góra | Western region | 51.65917 | 16.56453 | 1.50 |
| 2017 | 10 | Autumn | Lubuskie | Gorzów Wielkopolski | Western expansion zone | 52.76383 | 15.23103 | 0.50 |
| 2017 | 10 | Autumn | Lubuskie | Murzynowo | Western expansion zone | 52.642065. | 15.44346 | 0.00 |
| 2017 | 10 | Autumn | Lubuskie | Skwierzyna | Western expansion zone | 52.56630 | 15.52442 | 0.00 |
| 2017 | 10 | Autumn | Lubuskie | Stare Polichno | Western expansion zone | 52.70771 | 15.42598 | 0.00 |
| 2017 | 10 | Autumn | Wielkopolskie | Bystrzek | Western expansion zone | 52.08823 | 17.08858 | 0.00 |
| 2017 | 10 | Autumn | Wielkopolskie | Grodzisk Wielkopolski | Western expansion zone | 52.21680 | 16.36090 | 0.00 |
| 2017 | 10 | Autumn | Wielkopolskie | Kościan | Western expansion zone | 52.10445 | 16.63240 | 1.33 |
| 2017 | 10 | Autumn | Wielkopolskie | Kotowo | Western expansion zone | 52.22555 | 16.49109 | 0.00 |
| 2017 | 10 | Autumn | Wielkopolskie | Nowy Tomyśl | Western expansion zone | 52.30958 | 16.11307 | 3.60 |
| 2017 | 10 | Autumn | Wielkopolskie | Sroczewo | Western expansion zone | 52.09569 | 17.15269 | 0.00 |
| 2017 | 10 | Autumn | Zachodnio-pomorskie | Chojna | Western expansion zone | 53.12995 | 14.42094 | 2.33 |
| 2017 | 10 | Autumn | Zachodnio-pomorskie | Karsko | Western expansion zone | 52.90466 | 15.12033 | 0.00 |
| 2017 | 10 | Autumn | Zachodnio-pomorskie | Lubanowo | Western expansion zone | 53.12764 | 14.61369 | 0.00 |
| 2017 | 10 | Autumn | Zachodnio-pomorskie | Lubiczyn | Western expansion zone | 53.12995 | 14.42094 | 0.80 |
| 2017 | 10 | Autumn | Zachodnio-pomorskie | Łubianka | Western expansion zone | 52.88638 | 15.18776 | 0.00 |
| 2017 | 10 | Autumn | Zachodnio-pomorskie | Łubianka 2 | Western expansion zone | 52.89080 | 15.17779 | 0.00 |
| 2017 | 10 | Autumn | Zachodnio-pomorskie | Mieszkowice | Western expansion zone | 52.78966 | 14.51341 | 6.00 |
| 2017 | 10 | Autumn | Zachodnio-pomorskie | Myślibórz | Western expansion zone | 52.90904 | 14.92335 | 2.25 |
| 2017 | 10 | Autumn | Zachodnio-pomorskie | Rów | Western expansion zone | 52.97834 | 14.72285 | 2.17 |
| 2017 | 10 | Autumn | Dolnośląskie | Korzeńsko | Western expansion zone | 51.53542 | 16.87672 | 4.00 |
| 2017 | 10 | Autumn | Dolnośląskie | Niezgoda | Western expansion zone | 51.51415 | 17.04281 | 0.17 |
| 2017 | 10 | Autumn | Dolnośląskie | Postolin | Western expansion zone | 51.47224 | 17.24656 | 0.00 |
| 2017 | 10 | Autumn | Dolnośląskie | Wilków | Western expansion zone | 51.69495 | 16.20960 | 1.00 |
| 2018 | 3 | Spring | Mazowieckie | Adamowice | Eastern region | 51.94149 | 20.47620 | 3.25 |
| 2018 | 3 | Spring | Mazowieckie | Korabiewice | Eastern region | 51.95094 | 20.42969 | 42.00 |
| 2018 | 3 | Spring | Mazowieckie | Stoski | Eastern region | 52.40923 | 21.50913 | 48.00 |
| 2018 | 3 | Spring | Mazowieckie | Wręcza/Mszczonów | Eastern region | 51.97571 | 20.49028 | 26.00 |
| 2018 | 3 | Spring | Mazowieckie | Żyrardów | Eastern region | 52.07438 | 20.46676 | 59.00 |
| 2018 | 4 | Spring | Kujawsko-pomorskie | Boża Wola | Eastern expansion zone | 52.48200 | 19.25470 | 24.00 |
| 2018 | 4 | Spring | Kujawsko-pomorskie | Dębniaki | Eastern expansion zone | 52.54940 | 19.18160 | 0.86 |
| 2018 | 4 | Spring | Kujawsko-pomorskie | Lubianka 2 | Eastern expansion zone | 52.89080 | 15.17779 | 0.00 |
| 2018 | 4 | Spring | Kujawsko-pomorskie | Sokotowo-Parcele | Eastern expansion zone | 52.56689 | 18.91067 | 0.00 |
| 2018 | 4 | Spring | Łódzkie | Bełchów1 | Eastern expansion zone | 52.04000 | 20.05900 | 0.38 |
| 2018 | 4 | Spring | Łódzkie | Brończyń | Eastern expansion zone | 51.63510 | 18.38530 | 0.00 |
| 2018 | 4 | Spring | Łódzkie | Chwalborzyce | Eastern expansion zone | 52.06810 | 18.83970 | 2.00 |
| 2018 | 4 | Spring | Łódzkie | Drzewica | Eastern expansion zone | 51.45130 | 20.45910 | 2.25 |
| 2018 | 4 | Spring | Łódzkie | Jakubów | Eastern expansion zone | 51.58982 | 20.03771 | 0.00 |
| 2018 | 4 | Spring | Łódzkie | Kaczka | Eastern expansion zone | 51.55181 | 20.03962 | 3.25 |
| 2018 | 4 | Spring | Łódzkie | Klemetów | Eastern expansion zone | 51.90900 | 18.94080 | 0.00 |
| 2018 | 4 | Spring | Łódzkie | Krępa | Eastern expansion zone | 52.02624 | 19.83579 | 1.50 |
| 2018 | 4 | Spring | Łódzkie | Lubianków | Eastern expansion zone | 51.95410 | 19.79490 | 1.00 |
| 2018 | 4 | Spring | Łódzkie | Lubień | Eastern expansion zone | 52.01540 | 19.22740 | 2.50 |
| 2018 | 4 | Spring | Łódzkie | Lubochnia Górki | Eastern expansion zone | 51.59450 | 20.03060 | 0.00 |
| 2018 | 4 | Spring | Łódzkie | Łochów 2 | Eastern expansion zone | 51.74892 | 20.07967 | 5.00 |
| 2018 | 4 | Spring | Łódzkie | Łyszkowice | Eastern expansion zone | 51.98622 | 19.91374 | 0.50 |
| 2018 | 4 | Spring | Łódzkie | Niewiadów | Eastern expansion zone | 51.62195 | 19.90934 | 0.00 |
| 2018 | 4 | Spring | Łódzkie | Nowe Rowiska | Eastern expansion zone | 51.90006 | 20.12697 | 4.00 |
| 2018 | 4 | Spring | Łódzkie | Opoczno | Eastern expansion zone | 51.36880 | 20.27640 | 0.00 |
| 2018 | 4 | Spring | Łódzkie | Ozorków | Eastern expansion zone | 51.97310 | 19.29110 | 0.00 |
| 2018 | 4 | Spring | Łódzkie | Rawa Mazowiecka | Eastern expansion zone | 51.75236 | 20.24912 | 0.88 |
| 2018 | 4 | Spring | Łódzkie | Rawa Mazowiecka 2 | Eastern expansion zone | 51.76399 | 20.27001 | 0.00 |
| 2018 | 4 | Spring | Łódzkie | Sobota | Eastern expansion zone | 52.11127 | 19.68434 | 1.83 |
| 2018 | 4 | Spring | Łódzkie | Sójki | Eastern expansion zone | 52.29120 | 19.38200 | 0.00 |
| 2018 | 4 | Spring | Łódzkie | Sulejów | Eastern expansion zone | 51.35930 | 19.88040 | 0.00 |
| 2018 | 4 | Spring | Łódzkie | Teofilów | Eastern expansion zone | 51.52420 | 20.19550 | 12.67 |
| 2018 | 4 | Spring | Łódzkie | Warta | Eastern expansion zone | 51.71480 | 18.64810 | 0.00 |
| 2018 | 4 | Spring | Łódzkie | Władysławów | Eastern expansion zone | 51.97700 | 19.52510 | 1.50 |
| 2018 | 4 | Spring | Łódzkie | Wola Błędowa | Eastern expansion zone | 51.93380 | 19.63290 | 0.00 |
| 2018 | 4 | Spring | Łódzkie | Wola Naropińska | Eastern expansion zone | 51.75388 | 20.07879 | 0.00 |
| 2018 | 4 | Spring | Świętokrzyskie | Trębowiec Krupów | Eastern expansion zone | 51.17530 | 21.07630 | 1.40 |
| 2018 | 4 | Spring | Wielkopolskie | Sławoszewek | Eastern expansion zone | 52.39350 | 18.18320 | 5.00 |
| 2018 | 4 | Spring | Wielkopolskie | Sokołowo | Eastern expansion zone | 52.26870 | 18.68430 | 0.00 |
| 2018 | 4 | Spring | Mazowieckie | Białobrzegi | Eastern region | 51.66445 | 20.94700 | 8.20 |
| 2018 | 4 | Spring | Mazowieckie | Budki żelazowe | Eastern region | 52.28060 | 20.32990 | 1.40 |
| 2018 | 4 | Spring | Mazowieckie | Drezno | Eastern region | 51.21080 | 21.61870 | 4.00 |
| 2018 | 4 | Spring | Mazowieckie | Gołędzin | Eastern region | 51.41240 | 20.97299 | 2.00 |
| 2018 | 4 | Spring | Mazowieckie | Gulinek | Eastern region | 51.46220 | 21.00130 | 16.50 |
| 2018 | 4 | Spring | Mazowieckie | Jaworek | Eastern region | 52.42710 | 19.52470 | 15.50 |
| 2018 | 4 | Spring | Mazowieckie | Kociołki | Eastern region | 51.54245 | 21.55774 | 1.57 |
| 2018 | 4 | Spring | Mazowieckie | Korzeń Królewski | Eastern region | 52.43470 | 19.62590 | 0.88 |
| 2018 | 4 | Spring | Mazowieckie | Kowala | Eastern region | 51.32423 | 21.07455 | 11.00 |
| 2018 | 4 | Spring | Mazowieckie | Krakowa Góra | Eastern region | 51.32158 | 20.66409 | 5.78 |
| 2018 | 4 | Spring | Mazowieckie | Maruszów | Eastern region | 51.08632 | 21.61987 | 0.00 |
| 2018 | 4 | Spring | Mazowieckie | Owadów | Eastern region | 51.48825 | 21.17526 | 23.00 |
| 2018 | 4 | Spring | Mazowieckie | Pasztowa Wola | Eastern region | 51.08832 | 21.22927 | 7.20 |
| 2018 | 4 | Spring | Mazowieckie | Plecewice | Eastern region | 52.28498 | 20.27473 | 4.10 |
| 2018 | 4 | Spring | Mazowieckie | Podsuliszka | Eastern region | 51.24096 | 21.18053 | 1.67 |
| 2018 | 4 | Spring | Mazowieckie | Stanisławów | Eastern region | 51.24609 | 20.78781 | 2.00 |
| 2018 | 4 | Spring | Mazowieckie | Wieniawa | Eastern region | 51.34750 | 20.79506 | 4.33 |
| 2018 | 4 | Spring | Mazowieckie | Wierzbica | Eastern region | 51.27450 | 21.10530 | 7.50 |
| 2018 | 4 | Spring | Mazowieckie | Zwoleń | Eastern region | 51.33543 | 21.59029 | 10.75 |
| 2018 | 4 | Spring | Dolnośląskie | Głogów | Western region | 51.67890 | 16.11210 | 0.86 |
| 2018 | 4 | Spring | Dolnośląskie | Kawice | Western region | 51.24540 | 16.44950 | 20.50 |
| 2018 | 4 | Spring | Lubuskie | Kowalewo | Western region | 51.72830 | 16.31880 | 16.50 |
| 2018 | 4 | Spring | Lubuskie | Leśna Góra | Western region | 52.02650 | 15.61220 | 11.20 |
| 2018 | 4 | Spring | Lubuskie | Nowogród Bobrzański | Western region | 51.79420 | 15.24960 | 5.20 |
| 2018 | 4 | Spring | Lubuskie | Sulechów | Western region | 52.07050 | 15.60520 | 1.33 |
| 2018 | 4 | Spring | Lubuskie | Szprotawy | Western region | 51.56430 | 15.55720 | 2.80 |
| 2018 | 4 | Spring | Lubuskie | Trzciel | Western region | 52.37187 | 15.86673 | 5.71 |
| 2018 | 4 | Spring | Wielkopolskie | Leszno | Western region | 51.81670 | 16.53910 | 4.20 |
| 2018 | 4 | Spring | Dolnośląskie | Czernina Dolna | Western region | 51.73960 | 16.56510 | 5.75 |
| 2018 | 4 | Spring | Dolnośląskie | Biedaszków | Western expansion zone | 51.59280 | 17.61830 | 0.00 |
| 2018 | 4 | Spring | Dolnośląskie | Biedaszków Mały | Western expansion zone | 51.39966 | 17.09868 | 4.67 |
| 2018 | 4 | Spring | Lubuskie | Bobowicko | Western expansion zone | 52.43990 | 15.64940 | 0.43 |
| 2018 | 4 | Spring | Lubuskie | Gorzów Wielkopolski | Western expansion zone | 52.71680 | 15.30750 | 0.25 |
| 2018 | 4 | Spring | Lubuskie | Kłodawa | Western expansion zone | 52.77430 | 15.23130 | 0.00 |
| 2018 | 4 | Spring | Lubuskie | Murzynowo | Western expansion zone | 52.65045 | 15.46205 | 0.00 |
| 2018 | 4 | Spring | Lubuskie | Skwierzyna | Western expansion zone | 52.58640 | 15.51270 | 0.20 |
| 2018 | 4 | Spring | Lubuskie | Skwierzyna 2 | Western expansion zone | 52.60350 | 15.49850 | 0.00 |
| 2018 | 4 | Spring | Wielkopolskie | Bonikowo | Western expansion zone | 52.11619 | 16.63196 | 0.13 |
| 2018 | 4 | Spring | Wielkopolskie | Bugaj | Western expansion zone | 52.33900 | 18.69540 | 0.00 |
| 2018 | 4 | Spring | Wielkopolskie | Chojniki | Western expansion zone | 52.29160 | 16.12790 | 0.88 |
| 2018 | 4 | Spring | Wielkopolskie | Ciązeń | Western expansion zone | 52.21440 | 17.81300 | 0.00 |
| 2018 | 4 | Spring | Wielkopolskie | Czarna Wieś | Western expansion zone | 52.23000 | 16.22590 | 0.67 |
| 2018 | 4 | Spring | Wielkopolskie | Dulsk | Western expansion zone | 52.21270 | 17.70630 | 0.00 |
| 2018 | 4 | Spring | Wielkopolskie | Dulsk 2 | Western expansion zone | 52.21570 | 17.71520 | 0.00 |
| 2018 | 4 | Spring | Wielkopolskie | Kobylniki | Western expansion zone | 52.21550 | 16.32580 | 0.00 |
| 2018 | 4 | Spring | Wielkopolskie | Kościan | Western expansion zone | 52.10445 | 16.63240 | 0.00 |
| 2018 | 4 | Spring | Wielkopolskie | Książ Wielkopolski | Western expansion zone | 52.05850 | 17.23320 | 0.00 |
| 2018 | 4 | Spring | Wielkopolskie | Marcinkowo | Western expansion zone | 52.37740 | 18.57060 | 0.00 |
| 2018 | 4 | Spring | Wielkopolskie | Młodojew-Kolonia | Western expansion zone | 52.30180 | 17.93560 | 0.00 |
| 2018 | 4 | Spring | Wielkopolskie | Przygodzice | Western expansion zone | 51.58100 | 17.82310 | 0.00 |
| 2018 | 4 | Spring | Wielkopolskie | Stare Długie | Western expansion zone | 51.83230 | 16.44710 | 0.00 |
| 2018 | 4 | Spring | Wielkopolskie | Śrem | Western expansion zone | 52.08880 | 16.98710 | 0.33 |
| 2018 | 4 | Spring | Zachodniopomorskie | Banie | Western expansion zone | 53.09980 | 14.66880 | 0.25 |
| 2018 | 4 | Spring | Zachodniopomorskie | Dębno | Western expansion zone | 52.75050 | 14.69060 | 5.22 |
| 2018 | 4 | Spring | Zachodniopomorskie | Karsko | Western expansion zone | 52.90820 | 15.10330 | 0.00 |
| 2018 | 4 | Spring | Zachodniopomorskie | Myślibórz | Western expansion zone | 52.94152 | 14.87199 | 0.22 |
| 2018 | 4 | Spring | Zachodniopomorskie | Rurka | Western expansion zone | 52.99530 | 14.49230 | 4.50 |
| 2018 | 4 | Spring | Zachodniopomorskie | Tarnowo | Western expansion zone | 52.99290 | 14.82220 | 0.25 |
| 2018 | 4 | Spring | Dolnośląskie | Bożeń | Western expansion zone | 51.38870 | 16.63540 | 0.75 |
| 2018 | 4 | Spring | Dolnośląskie | Koczurki | Western expansion zone | 51.39100 | 17.00290 | 0.00 |
| 2018 | 4 | Spring | Dolnośląskie | Psary | Western expansion zone | 51.19400 | 17.03330 | 2.50 |
| 2018 | 4 | Spring | Dolnośląskie | Sułów | Western expansion zone | 51.48980 | 17.16650 | 0.00 |
| 2018 | 4 | Spring | Dolnośląskie | Wierzchowice Wielkie | Western expansion zone | 51.55975 | 16.60470 | 10.00 |
| 2018 | 4 | Spring | Dolnośląskie | Wrocław | Western expansion zone | 51.12810 | 16.86270 | 8.67 |
| 2018 | 4 | Spring | Dolnośląskie | Zakrzyce | Western expansion zone | 51.11830 | 16.78690 | 1.67 |
| 2018 | 10 | Autumn | Kujawsko-pomorskie | Dębniaki | Eastern expansion zone | 52.55046 | 19.18276 | 0.20 |
| 2018 | 10 | Autumn | Kujawsko-pomorskie | Izbica Kujawska | Eastern expansion zone | 52.41710 | 18.75100 | 0.00 |
| 2018 | 10 | Autumn | Łódzkie | Bełchów 2 | Eastern expansion zone | 52.01371 | 20.02561 | 0.08 |
| 2018 | 10 | Autumn | Łódzkie | Jeżów | Eastern expansion zone | 51.82881 | 19.98250 | 0.00 |
| 2018 | 10 | Autumn | Łódzkie | Koziołki | Eastern expansion zone | 51.87660 | 19.81590 | 0.00 |
| 2018 | 10 | Autumn | Łódzkie | Łochów 2 | Eastern expansion zone | 51.74892 | 20.07967 | 0.20 |
| 2018 | 10 | Autumn | Łódzkie | Łyszkowice | Eastern expansion zone | 51.97282 | 19.90143 | 0.00 |
| 2018 | 10 | Autumn | Łódzkie | Mroczków Gościnny | Eastern expansion zone | 51.38020 | 20.37560 | 0.00 |
| 2018 | 10 | Autumn | Łódzkie | Rawa Mazowiecka | Eastern expansion zone | 51.76563 | 20.26384 | 0.00 |
| 2018 | 10 | Autumn | Łódzkie | Rogów | Eastern expansion zone | 51.80375 | 19.83176 | 0.00 |
| 2018 | 10 | Autumn | Łódzkie | Słomków | Eastern expansion zone | 51.95529 | 19.98709 | 0.10 |
| 2018 | 10 | Autumn | Łódzkie | Sługodzice | Eastern expansion zone | 51.49190 | 20.10760 | 0.33 |
| 2018 | 10 | Autumn | Łódzkie | Tomaszów Mazowiecki | Eastern expansion zone | 51.52180 | 20.05790 | 0.50 |
| 2018 | 10 | Autumn | Świętokrzyskie | Starachowice | Eastern expansion zone | 51.06400 | 21.04170 | 0.00 |
| 2018 | 10 | Autumn | Wielkopolskie | Orzechowo | Eastern expansion zone | 52.10850 | 17.47640 | 0.00 |
| 2018 | 10 | Autumn | Wielkopolskie | Przykona | Eastern expansion zone | 51.98150 | 18.60730 | 0.00 |
| 2018 | 10 | Autumn | Wielkopolskie | Rosocha | Eastern expansion zone | 52.24890 | 18.16030 | 0.00 |
| 2018 | 10 | Autumn | Wielkopolskie | Sławoszewek | Eastern expansion zone | 52.39350 | 18.18320 | 0.00 |
| 2018 | 10 | Autumn | Wielkopolskie | Janowice | Eastern expansion zone | 52.32100 | 18.55710 | 0.00 |
| 2018 | 10 | Autumn | Wielkopolskie | Janowice 2 | Eastern expansion zone | 52.31720 | 18.54800 | 0.00 |
| 2018 | 10 | Autumn | Mazowieckie | Adamowice | Eastern region | 51.94149 | 20.47620 | 2.33 |
| 2018 | 10 | Autumn | Mazowieckie | Białobrzegi | Eastern region | 51.66445 | 20.94700 | 91.00 |
| 2018 | 10 | Autumn | Mazowieckie | Borowiec | Eastern region | 51.32530 | 20.67880 | 6.33 |
| 2018 | 10 | Autumn | Mazowieckie | Iłża | Eastern region | 51.15300 | 21.23630 | 0.75 |
| 2018 | 10 | Autumn | Mazowieckie | Korabiewice | Eastern region | 51.95094 | 20.42969 | 22.00 |
| 2018 | 10 | Autumn | Mazowieckie | Owadów | Eastern region | 51.48825 | 21.17526 | 48.00 |
| 2018 | 10 | Autumn | Mazowieckie | Pastwiska | Eastern region | 51.11840 | 21.21980 | 0.00 |
| 2018 | 10 | Autumn | Mazowieckie | Plecewice | Eastern region | 52.28593 | 20.27635 | 0.00 |
| 2018 | 10 | Autumn | Mazowieckie | Siekierki | Eastern region | 52.20404 | 21.08427 | 11.50 |
| 2018 | 10 | Autumn | Mazowieckie | Skoki | Eastern region | 52.39507 | 19.51830 | 4.25 |
| 2018 | 10 | Autumn | Mazowieckie | Stoski | Eastern region | 52.40923 | 21.50913 | 25.00 |
| 2018 | 10 | Autumn | Mazowieckie | Szydłowiec | Eastern region | 51.22030 | 20.87860 | 2.17 |
| 2018 | 10 | Autumn | Mazowieckie | Żyrardów | Eastern region | 52.07438 | 20.46676 | 5.00 |
| 2018 | 10 | Autumn | Dolnośląskie | Kawice | Western region | 51.22575 | 16.44067 | 26.00 |
| 2018 | 10 | Autumn | Lubuskie | Czciradz | Western region | 51.73510 | 15.63510 | 4.00 |
| 2018 | 10 | Autumn | Lubuskie | Leśna Góra | Western region | 52.02650 | 15.61220 | 0.00 |
| 2018 | 10 | Autumn | Lubuskie | Nowa Wieś | Western region | 51.83820 | 16.66470 | 4.40 |
| 2018 | 10 | Autumn | Lubuskie | Nowogród Bobrzański | Western region | 51.79420 | 15.24960 | 3.60 |
| 2018 | 10 | Autumn | Lubuskie | Siedlisko | Western region | 51.78540 | 15.83520 | 16.00 |
| 2018 | 10 | Autumn | Lubuskie | Świebodzin | Western region | 52.22820 | 15.52850 | 17.00 |
| 2018 | 10 | Autumn | Dolnośląskie | Czeszów | Western expansion zone | 51.37780 | 17.24220 | 0.17 |
| 2018 | 10 | Autumn | Lubuskie | Goraj | Western expansion zone | 52.56170 | 15.80170 | 0.00 |
| 2018 | 10 | Autumn | Lubuskie | Gorzów Wlkp. | Western expansion zone | 52.74180 | 15.19850 | 1.25 |
| 2018 | 10 | Autumn | Lubuskie | Ściechów | Western expansion zone | 52.81990 | 14.95190 | 0.00 |
| 2018 | 10 | Autumn | Lubuskie | Skwierzyna | Western expansion zone | 52.59630 | 15.51910 | 0.00 |
| 2018 | 10 | Autumn | Wielkopolskie | Chmielinko | Western expansion zone | 52.40400 | 16.16590 | 4.00 |
| 2018 | 10 | Autumn | Wielkopolskie | Długie Nowe | Western expansion zone | 51.82910 | 16.24180 | 78.00 |
| 2018 | 10 | Autumn | Wielkopolskie | Godurowo | Western expansion zone | 51.89620 | 17.12640 | 0.00 |
| 2018 | 10 | Autumn | Wielkopolskie | Jarocin | Western expansion zone | 51.98300 | 17.53530 | 0.00 |
| 2018 | 10 | Autumn | Wielkopolskie | Kościan | Western expansion zone | 52.10445 | 16.63240 | 1.00 |
| 2018 | 10 | Autumn | Wielkopolskie | Łowyń | Western expansion zone | 52.50270 | 15.91380 | 0.00 |
| 2018 | 10 | Autumn | Wielkopolskie | Niepart | Western expansion zone | 51.70440 | 17.00250 | 0.00 |
| 2018 | 10 | Autumn | Wielkopolskie | Nowy Młyn | Western expansion zone | 52.14845 | 16.11387 | 9.00 |
| 2018 | 10 | Autumn | Wielkopolskie | Nowy Tomyśl | Western expansion zone | 52.30660 | 16.13560 | 5.00 |
| 2018 | 10 | Autumn | Wielkopolskie | Śrem | Western expansion zone | 52.08880 | 16.98710 | 1.17 |
| 2018 | 10 | Autumn | Wielkopolskie | Trzebosz | Western expansion zone | 51.66830 | 16.73180 | 0.00 |
| 2018 | 10 | Autumn | Wielkopolskie | Wola Rychwalska | Western expansion zone | 52.07810 | 18.22030 | 0.00 |
| 2018 | 10 | Autumn | Wielkopolskie | Wytomyśl | Western expansion zone | 52.37520 | 16.17590 | 0.00 |
| 2018 | 10 | Autumn | Zachodniopomorskie | Banie | Western expansion zone | 53.09230 | 14.65070 | 1.50 |
| 2018 | 10 | Autumn | Zachodniopomorskie | Dalsze | Western expansion zone | 52.87940 | 14.85240 | 4.50 |
| 2018 | 10 | Autumn | Zachodniopomorskie | Krzemlin | Western expansion zone | 53.07110 | 14.88240 | 0.00 |
| 2018 | 10 | Autumn | Zachodniopomorskie | Otanów | Western expansion zone | 52.99750 | 14.87010 | 0.33 |
| 2018 | 10 | Autumn | Zachodniopomorskie | Rurka | Western expansion zone | 52.99530 | 14.49230 | 14.00 |
| 2018 | 10 | Autumn | Zachodniopomorskie | Tetyń | Western expansion zone | 53.04750 | 14.76340 | 0.75 |
| 2018 | 10 | Autumn | Dolnośląskie | Góra Wąsowa | Western expansion zone | 51.58330 | 16.69650 | 0.00 |
| 2018 | 10 | Autumn | Dolnośląskie | Jawor | Western expansion zone | 51.51490 | 16.64430 | 2.17 |
| 2018 | 10 | Autumn | Dolnośląskie | Niezgoda | Western expansion zone | 51.51200 | 17.05570 | 0.00 |
| 2018 | 10 | Autumn | Dolnośląskie | Poligon Cienin | Western expansion zone | 51.20000 | 17.08910 | 28.00 |
| 2018 | 10 | Autumn | Dolnośląskie | Sułów | Western expansion zone | 51.49340 | 17.15330 | 0.00 |
| 2018 | 10 | Autumn | Dolnośląskie | Wrocław | Western expansion zone | 51.15010 | 16.82920 | 14.67 |
| 2018 | 10 | Autumn | Dolnośląskie | Wyjeździec Wielki | Western expansion zone | 51.41430 | 17.10255 | 0.00 |
